# Defining and characterizing the critical transition state prior to the type 2 diabetes disease

**DOI:** 10.1371/journal.pone.0180937

**Published:** 2017-07-07

**Authors:** Bo Jin, Rui Liu, Shiying Hao, Zhen Li, Chunqing Zhu, Xin Zhou, Pei Chen, Tianyun Fu, Zhongkai Hu, Qian Wu, Wei Liu, Daowei Liu, Yunxian Yu, Yan Zhang, Doff B. McElhinney, Yu-Ming Li, Devore S Culver, Shaun T. Alfreds, Frank Stearns, Karl G. Sylvester, Eric Widen, Xuefeng B. Ling

**Affiliations:** 1 HBI Solutions Inc., Palo Alto, California, United States of America; 2 Stanford University, Stanford, California, United States of America; 3 School of Mathematics, South China University of Technology, Guangzhou, China; 4 Clinical and Translational Research Program, Betty Irene Moore Children's Heart Center, Lucile Packard Children’s Hospital, Stanford, California, United States of America; 5 School of Electrical Engineering, Southeast University, Nanjing, China; 6 Tianjin Key Laboratory of Cardiovascular Remodeling and Target Organ Injury, Pingjin Hospital Heart Center, Tianjin, China; 7 School of Computer Science and Engineering, South China University of Technology, Guangzhou, China; 8 China Electric Power Research Institute, Beijing, China; 9 School of Medicine, Zhejiang University, Hangzhou, China; 10 Department of Oncology, the First Hospital of Shijiazhuang, Shijiazhuang, Hebei, China; 11 HealthInfoNet, Portland, Maine, United States of America; 12 Health Care Big Data Center, School of Public Health, Zhejiang University, Hangzhou, China; Shanghai Diabetes Institute, CHINA

## Abstract

**Background:**

Type 2 diabetes mellitus (T2DM), with increased risk of serious long-term complications, currently represents 8.3% of the adult population. We hypothesized that a critical transition state prior to the new onset T2DM can be revealed through the longitudinal electronic medical record (EMR) analysis.

**Method:**

We applied the transition-based network entropy methodology which previously identified a dynamic driver network (DDN) underlying the critical T2DM transition at the tissue molecular biological level. To profile pre-disease phenotypical changes that indicated a critical transition state, a cohort of 7,334 patients was assembled from the Maine State Health Information Exchange (HIE). These patients all had their first confirmative diagnosis of T2DM between January 1, 2013 and June 30, 2013. The cohort’s EMRs from the 24 months preceding their date of first T2DM diagnosis were extracted.

**Results:**

Analysis of these patients’ pre-disease clinical history identified a dynamic driver network (DDN) and an associated critical transition state six months prior to their first confirmative T2DM state.

**Conclusions:**

This 6-month window before the disease state provides an early warning of the impending T2DM, warranting an opportunity to apply proactive interventions to prevent or delay the new onset of T2DM.

## Introduction

Type 2 diabetes mellitus (T2DM) is a metabolic disorder characterized by hyperglycemia caused by either or both insulin resistance or insufficient insulin production. People with T2DM are at high risk for several serious health complications including cardiovascular diseases, blindness, kidney failure, limb amputations, premature death, fractures, frailty, depression, and cognitive decline. Because of the shifting age structure of the global population and the trend of urbanization and lifestyle changes in developing countries, the prevalence of diabetes among adults, 422 million worldwide in 2016, nearly doubles from 1980 to 2016 [1]. The total estimated economic impact of those diagnosed with diabetes in the US in 2012 was $245 billion, a cost that included $176 billion in direct medical costs and $69 billion in reduced productivity [2].

T2DM can often be prevented or delayed by maintaining a healthy life style, including a normal body weight, engaging in physical exercise smoking cessation, and consuming a healthful diet [3, 4]. A meta-analysis reported that 3-month lifestyle interventions decreased the risk for diabetes from the end of intervention up to 10 years later [5]. Additionally, a modifiable disease inflection point was hypothesized during the gradual progression to chronic diseases [6–11] after which the return to the pre-disease state is not possible. Therefore, it would be of high health priority to develop an early warning system for T2DM such that an opportunity window of greater than three months prior to the disease state shall warrant an effective intervention for the preventive care or the implementation of interventions to slow down the T2DM progression.

We hypothesized that complex diseases progress through three states, i.e., a normal state, a pre-disease state (or a critical transition state), and a disease state [12–15]. From population health point of view, it is essential to identify the pre-disease state to apply effective intervention, thus, qualitative deterioration can be managed. For many complex diseases, however, it is a difficult task to predict a pre-disease state because the state of the system may change little before the bifurcation point or the critical transition is reached, namely, there may be little difference between the normal and pre-disease states. A pre-disease state can be considered as a limit of the normal state but a disease state is different from the normal state. This is also why diagnoses based on traditional biomarkers or static measurements fail to distinguish a pre-disease state from a normal state. Additionally complexity of diseases, including T2DM, can involve thousands of genetic factors (e.g., SNPs and CNVs), epigenetic factors (e.g., methylation and acetylation), and different worldwide environment exposures. To resolve this problem, we piloted a novel model-free method to detect early warning signals of diseases [12–15]. Its theoretical foundation is based on dynamical driver network (DDN), which is also called as the driver (or leading) network of the disease because components in DDN drive the whole system from one state (e.g. normal state) to another (e.g. disease state). DDN can not only uncover significant signals among noises the emergence of the critical transitions for the commitment of disease progression, but can also help the formulation of discovery hypotheses down to genetic and biochemical pathways. However, DDN itself cannot simply be recognized as a causal network of T2DM critical transition.

We applied through the application of the Transition Network Entropy (TNE) and DDN methods to T2DM disease transition analysis with high throughput genomic datasets. Specifically, based on the temporal-spatial gene expression data of T2DM, we identified tissue-specific DDNs corresponding to the critical transitions during T2DM development and progression. Our hypothesis of T2DM critical transition state at the tissue molecular level was validated during disease deterioration and characterized as responses to insulin resistance and serious inflammation. Based on the functional and network analysis on pathogenic molecular mechanism of T2DM, we showed that most of DDN genes, particularly the core ones, tended to locate at the biological pathways upstream of T2DM pathophysiology, which implied that DDN-identified genes act as the causal factors rather than the consequence to drive the downstream molecules to change their transcriptional activities. This finding validated our theoretical prediction of DDN as the driver network.

An improved understanding of disease at the system medicine level is anticipated to result from the big data analytics of the such statewide or nationwide electronic medical records (EMRs), particularly when combined with natural language processing and extraction of the unstructured clinical note information [16–22]. Statewide health information exchange (HIE) is a secure, standardized electronic system where providers can share important patient health information for treatment purposes. Before the HIE implementation, there have been lack of accurate and complete electronic medical data, inability to access real-time information, and missed or late information. Patient records were stored in several physician offices, different hospital storage systems, and independent laboratories. Patient records were available only when provider offices are open or certain staff are available. Primary care physician was unaware or alerted late to rest results or hospital admission/discharge. With HIE, clinical medical records are now stored in one electronic location giving providers access to a complete patient view. Data can be accessible to providers in real-time. Once information is generated in native EMR, it’s in the HIE within minutes. Event alerts and notifications can be sent from HIE to care manager through email. In addition, with the advance of machine learning and artificial intelligence, HIE leadership started to focus on not merely delivering information, but also on putting the vast amount of data they collect to work on big data analytics helping hospitals, accountable care organizations and physician practices put the power of near real-time data to work on improving patient care.

A major advantage in the application of EMR data mining stems from parallel analysis of thousands of demographical/clinical parameters and events, like the analytics of high dimensional genomics experiments, allowing the efficient detection of discriminant clinical patterns for sensitive measure of pathophysiological status and monitoring altered disease progression. Changes in clinical status is then based on the detection of perturbations in the frequencies and measures of specific clinical events and laboratory test results involved in several key disease-related or other specific clinical pathways [16–22]. Thus, EMR analytics can reveal crucial information that is closely related to the disease progression or outcomes of targeted therapeutics.

In line with our previous work of diabetes transition analysis with genomic datasets, we hypothesize that a critical transition state of T2DM disease progression can be identified through EMR mining. The detection of the T2DM transition state using HIE EMRs prior to definitive T2DM onset would provide an opportunity window for effective interventions to prevent or delay the T2DM development. To test this hypothesis, we set to apply our transition-based network entropy model [23] to identify the critical transition state and the leading network of longitudinal clinical histories to device early warning system of impending T2DM for population health management.

## Result

### Hypothesis testing of the EMR based critical transition state prior to T2DM

The T2DM initiation dynamics before sudden deterioration is usually too complicated to be fully expressed mathematically in high dimensional spaces. The drastic or a qualitative transition in a local system or network, from a normal (i.e. disease-free) state to a disease state, corresponds to a so-called bifurcation point in dynamical systems theory [24, 25]. If the system is approaching a bifurcation point, it will eventually be constrained to a one- or two-dimensional space (i.e., the center manifold), in which a dynamical system can be expressed in a very simple form. This is the theoretical basis for developing a general indicator that can detect the T2DM critical transition.

**Fig 1:**
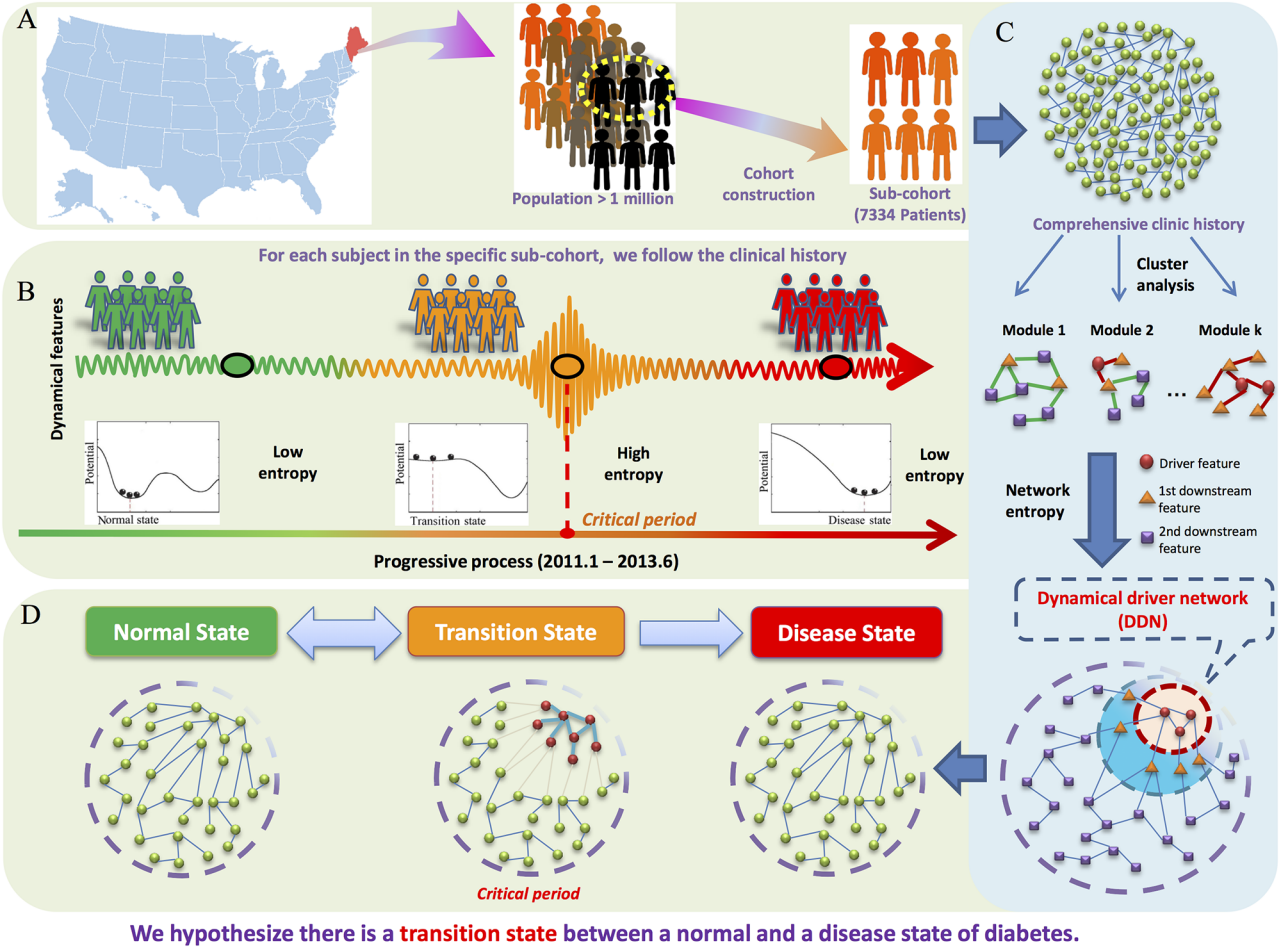
The schematic of dynamic driver network (DDN) based model integrated to the Maine Health Information Exchange workflow to characterize the critical transition state prior to the type 2 diabetes mellitus (T2DM) disease. (A) Based on clinical records of 1.3 million people from Maine State, USA, we carried out a population study and extracted a sub-cohort with 7,334 patients with the first T2DM confirmative diagnosis during the study period. (B) The progression of T2DM can be divided into three stages, i.e., the normal state with relatively low entropy, the transition state right before the critical transition with relatively high entropy, the disease state with relatively low entropy. The sharp increase of entropy is expected to characterize the transition state before getting into the disease state. (C) With a transition-based network entropy, the features can be classified into three layers, and the DDN can be obtained. Based on the dynamical characteristics (such as comprehensive clinic history, or time-course information) the cluster analysis suffices to separate the network into a few functional modules. Further analysis via network entropy aggregates these modules and identifies the DDN. (D) Employing the transition-based network entropy method, we succeed in presenting the existence of a transition state (orange) between a normal state (green) and a disease state (red). The network structure of features can be divided into two parts, the DDN, and other downstream features. The DDN provides the indicative warning signals to the sudden deterioration of diabetes. The map in the figure was created by Adobe Photoshop CS6 (https://helpx.adobe.com/x-productkb/policy-pricing/cs6-product-downloads.html).

As shown in Fig 1, we collected the comprehensive historical longitudinal EMR datasets from all of Maine’s HIE aggregated patients. A cohort of 7,334 patients with confirmatively diagnosis of T2DM between January 1, 2013 and June 30, 2013 was constructed (Fig 1A). We hypothesized three states during the T2DM progression can be characterized by clinical patterns in EMR: the normal state, the transition state, and the disease state (Fig 1B). To profile the critical transition state prior to T2DM, a feature correlation network with 33,403 features was analyzed using the network entropy algorithm (Fig 1C). The whole feature network was localized, and classified into three layers, which was calculated as the network entropy of the state change in a Markov process. Markov process and its ability to govern the dynamics of each node in the network was described in the following section. It was expected that there is a DDN with critical behaviors occurring when the system is in the transition state (Fig 1D).

### The critical transition detection prior to the first T2DM confirmative diagnosis

**Fig 2:**
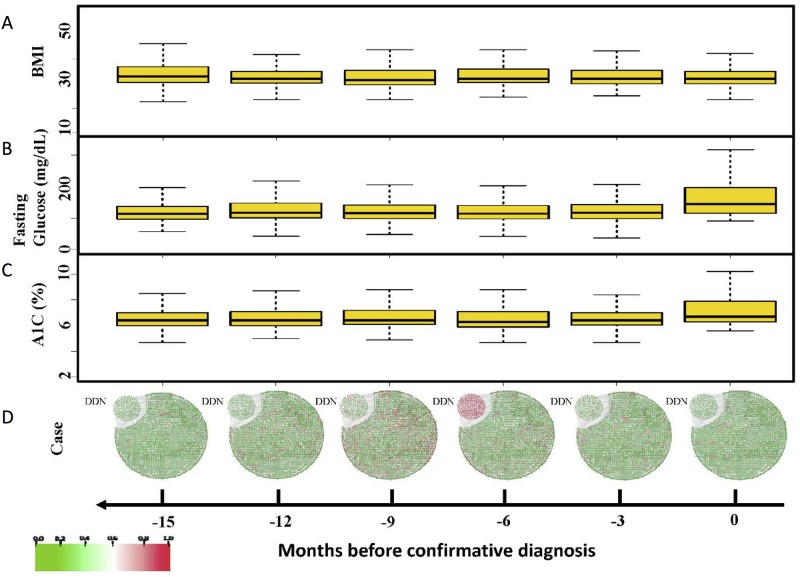
The comparison of dynamical evolution for the dynamic driver network (DDN) and the traditional biomarkers. (A) The trending of BMI illustrates no obvious increase tendency even when the patient is near the critical transition into type 2 diabetes mellitus (T2DM). (B) The glucose index remains a consistent value (less than 126 mg/dl) before the confirmative diagnosis (time point 0) with no indicative signal even when the patient is near the critical transition into T2DM. (C) The A1C index remains less than 6.5% before the confirmative diagnosis (time point 0) and does not change significantly even when the patient is near the critical transition into T2DM. (D) The evolution of DDN shows an early-warning signal can be detected 6 months before the diagnosis of T2DM.

As shown in Fig 1B, by applying the TNE method to the cohort’s clinical records, a DDN (n = 257 features) was identified to show the existence of the critical transition between a disease-free state and a T2DM disease state. Fig 2D presents the dynamical evolution in the whole feature network. For patients later developing T2DM, the DDN entropy becomes significantly high six months before the first confirmative diagnosis (Fig 2D), indicating a signal of the critical transition before the deterioration into T2DM. In contrast, there is no significant change in controls’ feature network dynamical progression (S1 Fig). When the deterioration is impending, these selected features form a special DDN sub-network, and make the first move from the normal state toward the disease state during the transition. DDN’s behavior validates our hypothesis that there is a transition state or pre-disease period between the normal and disease states. In the study population, the transition state occurred around 6 months before the T2DM confirmative state.

Fig 2A–2C show that the BMI, glucose, and A1C test results failed to identify the critical transition period for T2DM diagnosis on the same population. These results indicate that risk factors that are widely used by clinicians for T2DM diagnosis may not be able to serve as early-warning T2DM signals to alert patients prior to the confirmative diagnosis (i.e., prior to the time point 0 in Fig 2A–2C). It suggests that compared to the traditional diagnosis methods, the DDN-based approach is more effective in identifying the critical T2DM transition point.

### DDN’s functional characterization

**Fig 3:**
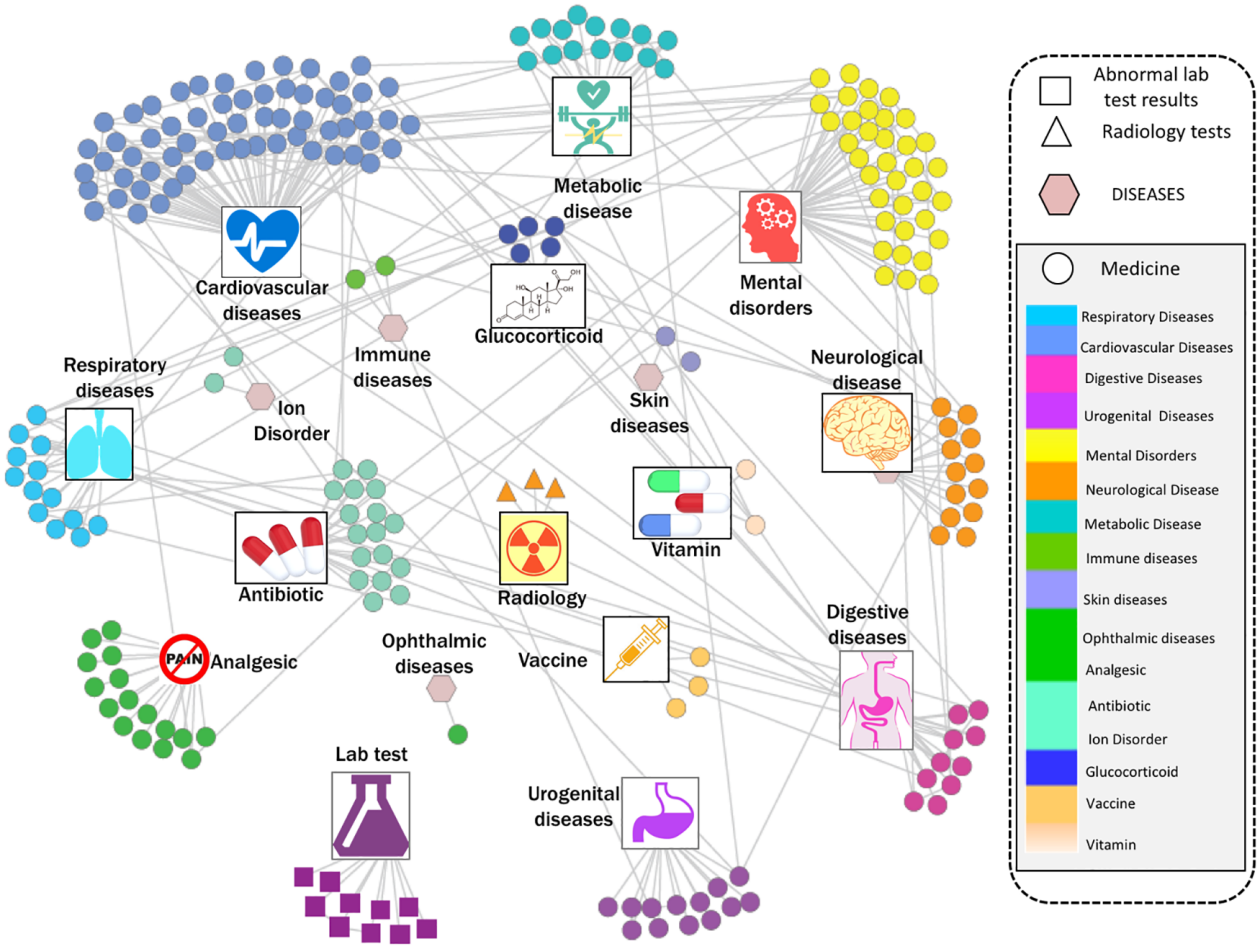
Modular structure of the dynamic driver network (DDN) features. The selected features of the DDN network can be classified into 16 subgroups of demographics, primary diagnosis, procedure, etc. Most features were found to be directly involving to the utilization of medicine to manage chronic diseases such as cardiovascular diseases and metabolic disease, which indicates the derived DDN is clinically reasonable for the critical transition identification of type 2 diabetes mellitus.

DDN features were further characterized for their association and relatedness to T2DM clinical development. The 257 selected features, which constructed the DDN, classify into abnormal lab test results, abnormal radiology test results, and medications. Medication features can be sub grouped by the associated primary diagnoses, procedures, or chronic conditions. Fig 3 summarizes the total 16 subgroups of features. Most features are correlated to the medication utilized in chronic diseases closely related to diabetes, such as cardiovascular diseases and metabolic disease. It indicates that the derived DDN is clinically reasonable for T2DM critical transition point identification, and thus highlights an opportunity to develop an early warning tool for clinical use based on the DDN method. Some features are related to abnormal lab test (n = 10 features) or the use of radiology tests (n = 3 features). These tests reflect the incubating risk factors, which may trigger the deterioration into T2DM.

### Clinical and resource utilization patterns during the transition to T2DM

**Fig 4:**
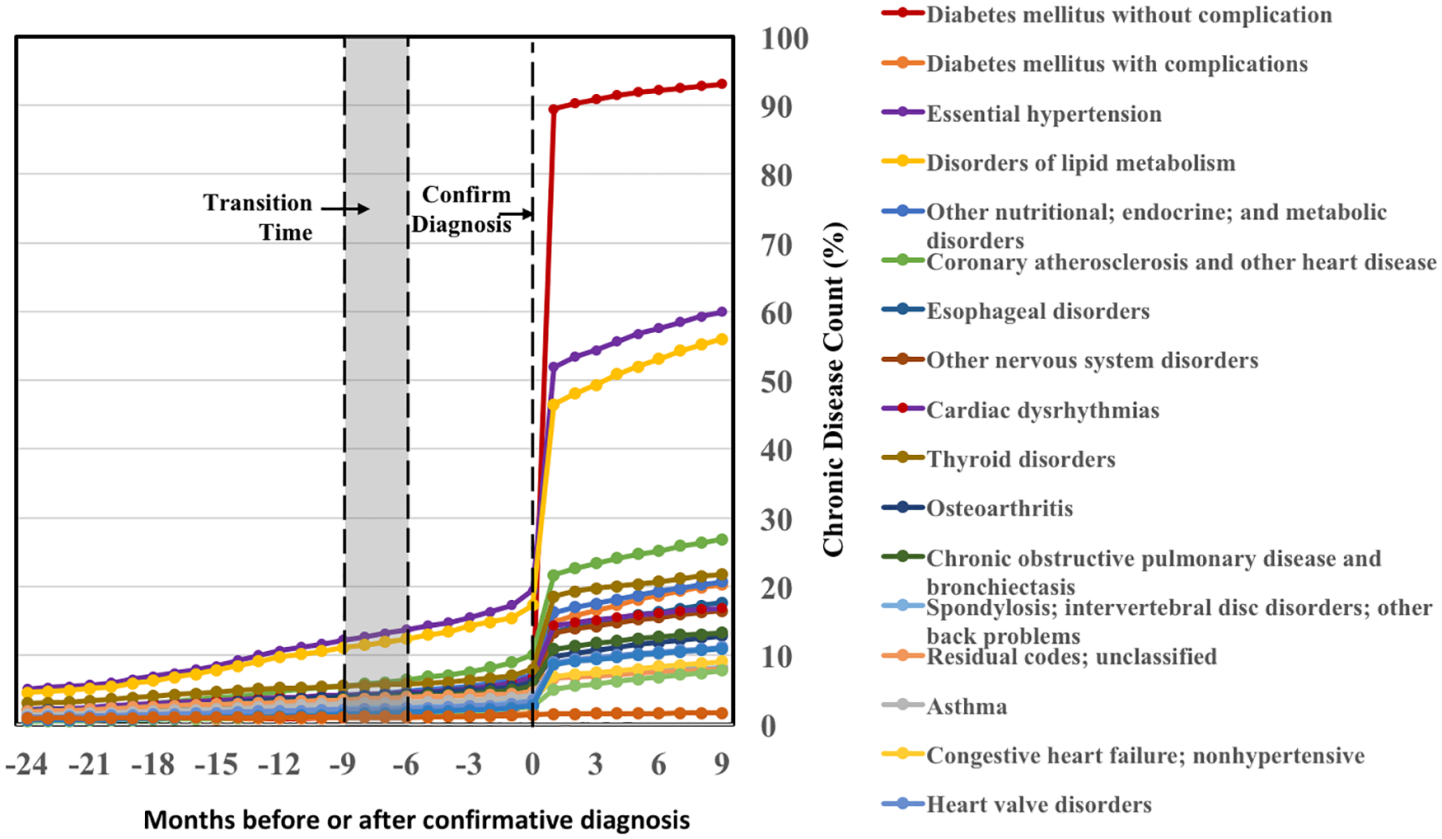
Trending of T2DM related chronic disease counts from 24 months prior to the diagnosis of T2DM to 6 months after the diagnosis of T2DM. The counts of diabetes mellitus without complication, the essential hypertension, and the disorders of lipid metabolism abruptly increase after the diagnosis was confirmed.

**Fig 5:**
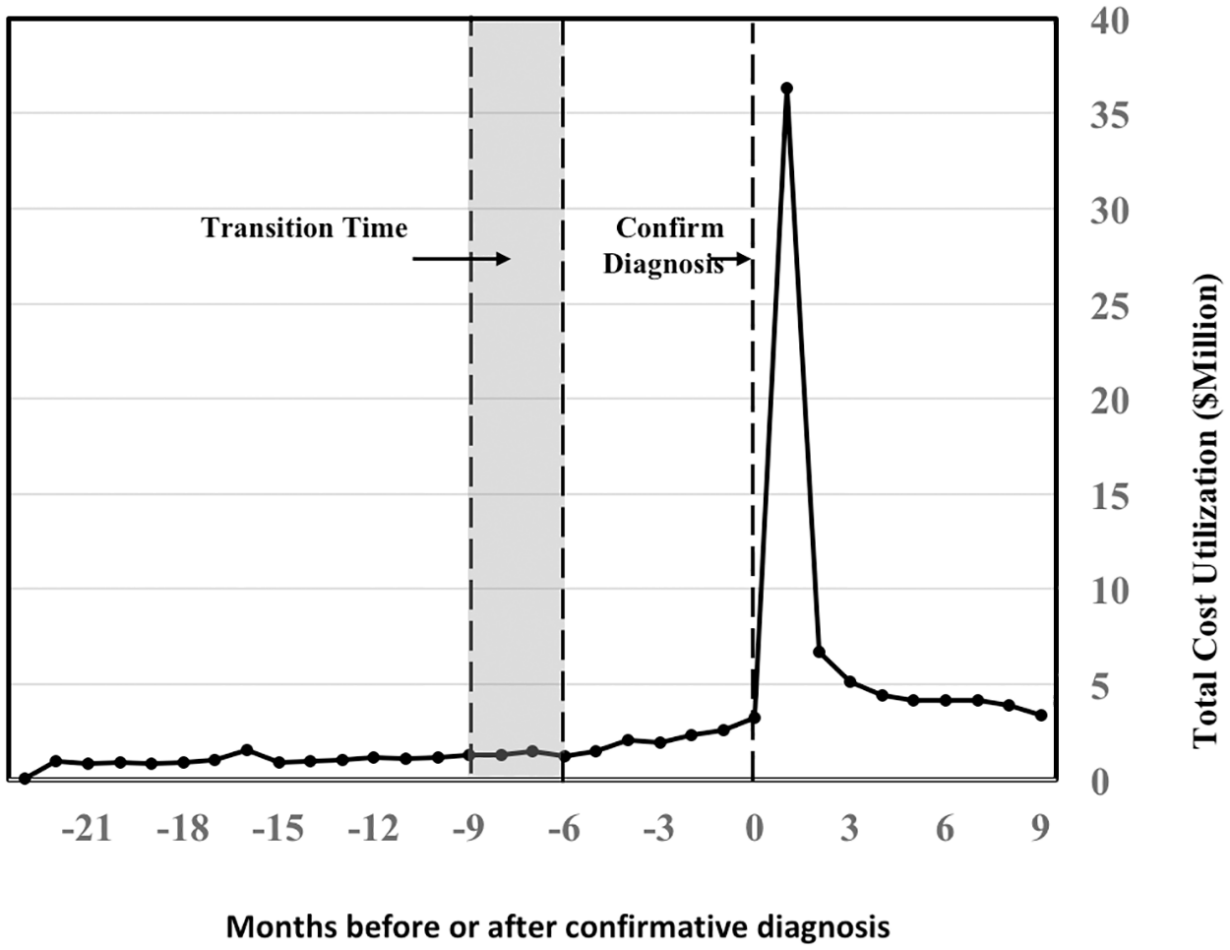
Trending of the total cost of utilization from 24 months prior to the diagnosis of type 2 diabetes mellitus (T2DM) to 6 months after the diagnosis of T2DM. The total cost reaches the peak rapidly after the confirmative diagnosis.

**Fig 6:**
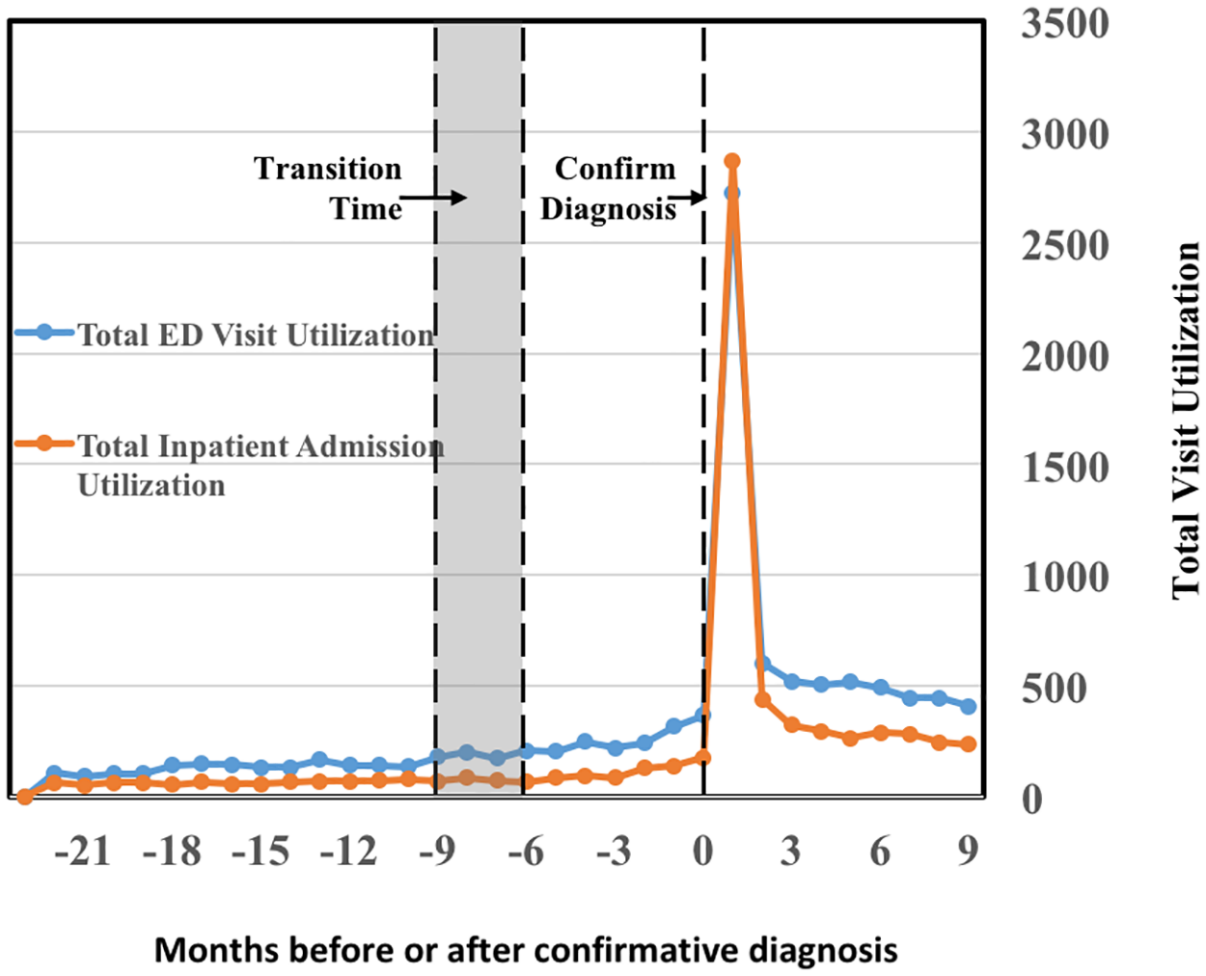
Trending of the total emergency department (ED) visit utilization and total inpatient admission utilization from 24 months prior to the diagnosis of type 2 diabetes mellitus (T2DM) to 6 months after the diagnosis of T2DM. The two curves both rise abruptly after confirmative diagnosis.

**Fig 7:**
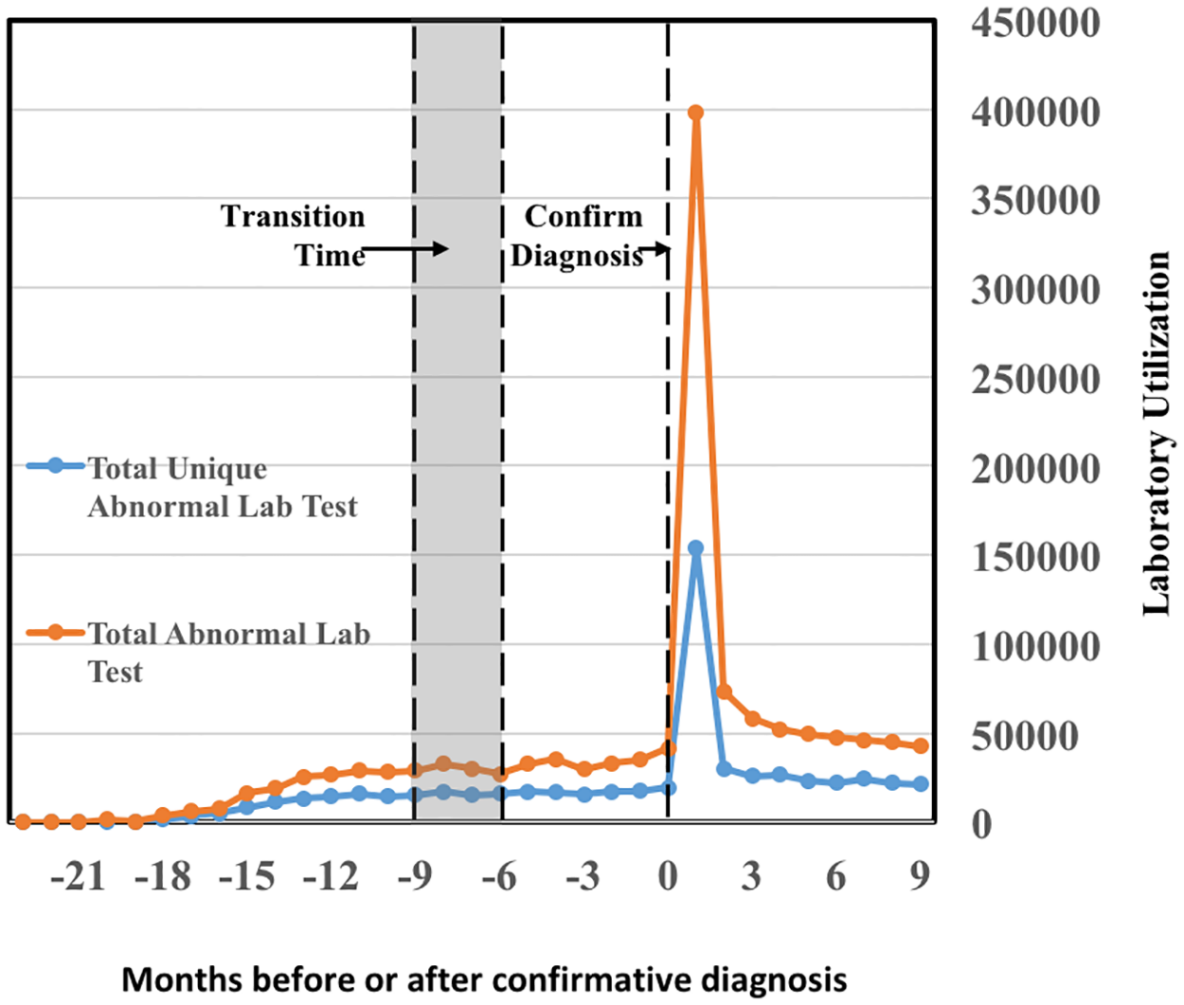
Trending of unique abnormal lab test volume and total abnormal lab test volume from 24 months prior to the diagnosis of type 2 diabetes mellitus (T2DM) to 6 months after the diagnosis of T2DM. Both volumes increase sharply after the confirmative diagnosis.

The TNE-based DDN method profiles and differentiates T2DM’s 3-state progression. We analyzed the changes of patients’ clinical outcomes and resource utilization from the normal state to the disease state (Figs 4–7). It shows that among the patients diagnosed as T2DM in the disease period, 60% had essential hypertension, and 50% had abnormalities of lipid metabolism (Fig 4). The presence of these chronic conditions makes T2DM a costly chronic disease. As shown in Figs 4–7, the associated costs and resource utilization including clinical visits and laboratory utilization abruptly increase rapidly until reaching a peak at one month after the diagnosis was confirmed, then quickly decline in the following months.

## Discussion

Consistent with our hypothesis, the results indicate that there is a critical transition that could be detected 6 months before the T2DM confirmative diagnosis. Unlike the detection of the disease state, the DDN enables the identification of an intermediate or transition state that generally has no clear metabolic abnormalities but with future trending of deterioration.

The DDN features discovered in this study may provide a repository of T2DM associative and/or disease driving clinical patterns underlying the progression of T2DM. This may help identify modifiable clinical features for proactive intervention for preventive care or to slow down the disease progression. Rather than using only a pre-defined set of clinical findings (such as IGT, IFG, A1C and BMI), we developed a mathematical approach of system medicine, focusing on the longitudinal evolution of each clinical feature and their relationships with each other during the procession of disease. The T2DM DDN network features (n = 257) can be divided into three subgroups: abnormal lab test results, abnormal radiology test results, and medications. Most of these features are highly correlated with T2DM or its complications, e.g. cardiovascular diseases and other metabolic diseases.

218/257 DDN features are medication prescriptions. Medication history can be used to gauge patient disease burden. Demographic characteristics (S2 Table) show that patients in transition to T2DM had significant chronic disease burden, including hypertension, heart problems and asthma, prior to the onset of T2DM, compared with controls. Such differences were reflected by medication prescriptions that comprised DDN. For example, Diltiazem, Metolazone, and Losartan can help to reduce risk of high blood pressure. Statin and Digoxin can help with cholesterol lowering and heart diseases. These chronic diseases and T2DM are interrelated diseases, and medications to treat these diseases may have either positive or negative impact on critical transition from latent to T2DM onset. Cholesterol drugs, including Lovastatin, Atorvastatin, and Crestor that were selected as DDN features, help to maintain balance between HDL and LDL cholesterol, which contributes to T2DM preventive care as low levels of HDL cholesterol are associated with elevated risk of T2DM [26]. Persistent medication history associated with elevated risk of T2DM illustrates the activation of the potential underlying clinical pathways responsible for the transition from latent to T2DM state. DDN medication features such as antibiotics (Levofloxacin, Fluoroquinolone, Azithromycin, and Nystatin), β blockers (Sotalol and Metoprolol), diuretics (Metolazone and Hydrochlorothiazide), and antidepressants (Amitriptyline, Citalopram, and Fluoxetine), can actually increase the risk of T2DM [27–30]. Both the positive and negative correlated medications in regard to T2DM disease transition collectively suggest that our findings suggest the clinical care utilities of the DDN features.

Like medication prescription, abnormal laboratory test results that were selected by DDN learning process also reflect clinical status of our cohort. Abnormal results of metabolic panel, cardiac panel, and urine culture are consistent with the cohort subjects’ comorbidities (S2 Table), including increased risk of having metabolic syndrome and obesity, heart problem, kidney diseases, and infection.

The cost and resource utilization among T2DM new onset cases primarily occurred and peaked shortly after the disease was confirmed. Such findings indicate that identification of transition state prior to T2DM may allow the timely initiation of interventions to reduce clinical resource utilization.

The DDN method is a generalized methodology for mining the dynamical information from the longitudinal clinical EMR patterns and identifying the transition state. This method was previously applied to genomics based diabetic disease progression analysis to identify the states of normal, critical transition, and new disease onset [13–15]. In this study, we applied this method to profile the EMR clinical patterns and identify the critical transition state of T2DM. The work constructs the first step towards the development of an early warning system of T2DM for population health.

Compared with other early warning methods to identify the critical transitions, the DDN method has several advantages. First, it is a more efficient method. Only the local information from a local network was involved in the calculation, which largely reduced the complexity. Second, by ignoring the nodes where the TNE changes slightly the noise in the calculation was essentially reduced, and the bias in identifying the critical transitions was avoided. Third, the conventional correlation-based methods analyzed the linear relationships between the nodes only, while the DDN method can depict the nonlinear relationships among the nodes. Fourth, the DDN features obtained by this method provide an approach to the underlying mechanisms during the progression of the disease, which would be very helpful in the development of the clinical treatment of the diseases.

T2DM is a progressive disease that is associated with a tremendous economic burden to both the U.S. health care system and the whole society. Reducing T2DM incidence and its complications through intensive lifestyle and pharmacologic interventions will result in better patient outcomes [31–33]. According to two clinical trials, a 3-month intensive lifestyle intervention in high-risk patients significantly reduced those diagnosed with diabetes during a 3- to 10-year period [34, 35]. The effectiveness of interventions, however, is largely dependent on the timing of their application relative to disease transitions. Our study shows that clinical profile of patients have detectable change prior to confirmative diagnosis of T2DM, compared with normal patients. Such change could happen during or even before the insulin resistance state when glucose level is still in a normal range. The clinical profile change, defined as critical transition state in this study, can be identified by our DDN algorithm. Identification of critical transition state of T2DM improves awareness of both providers and patients, and thus helps decision making on diet and life style interventions before the commitment of the T2DM progression. It warrants the timely interventions targeted to the high-risk patients prior to deterioration, which can device effective preventive care, improve the quality of disease management, and reduce the overall clinical resource utilization.

In our previous work, we demonstrated it is efficient method to detect both T1DM and T2DM using high dimensional genomics datasets [13–15]. This study integrated statewide HIE EMR longitudinal patterns and applied the TNE-based DDN method to uncover the disease transition state prior to the new onset T2DM. The DDN analytics with HIE real time EMR datasets can be generalized to apply to other complex chronic diseases. Our approach, to identify complex disease critical transition state as an early warning system and case-find high-risk patients statewide or nationwide in real time, can promise to translate the medicine of all complex chronic diseases.

We notice there are some limitations of this study. First, the average number of patient records prior to diagnosis of T2DM was 8.4, while 14% of patients had no patient record before diagnosis. These patients might have been diagnosed with T2DM somewhere out of HIE network, or have developed T2DM but without awareness. For those patients without EMR records of medication, laboratory test results, or chronic disease history, they may be bucketed into the baseline subgroup without commitment to T2DM progression. Second, data sources of this study are EMR datasets routinely collected by Maine HIE. Study population therefore are limited to those who had visited any of the care facilities participating HIE network. Adjustments would be needed when our statistical learning and the associated healthcare management protocols need to be transferred to populations in other geographical and/or cultural areas outside HIE network. Geographical, environmental, and racial disparity analyses may uncover additional T2DM critical transition features for T2DM population health.

## Methods

### Data availability

The work was performed under a business arrangement between HealthInfoNet (http://www.hinfonet.org), the operators of the Maine Health Information Exchange and HBI Solutions, Inc. (HBI) located in California. By business arrangement we mean HBI is a contracted vendor to HealthInfoNet (HIN), and HBI is under contract to deploy its proprietary applications and risk models on the HIN data for use by HIN members. HIN is a steward of the data on behalf of its members which includes health systems, hospitals, medical groups and federally qualified health centers. The data is owned by the HIN members, not HIN. HIN is responsible for security and access to its members' data and has established data service agreements (DSAs) restricting unnecessary exposure of information. HIN and its board (comprised from a cross section of its members) authorized the use of the de-identified data for this research, as the published research helps promote the value of the HIE and value to Maine residents.

HBI receives revenue for providing this service, which is performed remotely. HBI does not own or have access to the data outside of providing services to HIN. HIN manages and controls the data within its technology infrastructure. The research was conducted on HIN technology infrastructure, and the researchers accessed the de-identified data via secure remote methods. All data analysis and modeling for this manuscript was performed on HIN servers and data was accessed via secure connections controlled by HIN.

Access to the data used in the study requires secure connection to HIN servers and should be requested directly to HIN. Researchers may contact Phil Prefenno at pprofenno@hinfonet.org, (207) 541–4115 to request data. Data will be available upon request to all interested researchers. HIN agrees to provide access to the de-identified data on a per request basis to interested researchers. Future researchers will access the data through exact the same process as the authors of the manuscript.

### Ethics statement

The studies were performed in accordance with the Declaration of Helsinki and relevant local guidelines and regulations. This work was done under a business arrangement between HealthInfoNet (HIN), operator of the Maine HIE (a nonprofit organization to benefit the population health of the Maine State residents), and HBI Solutions, Inc (HBI). The data use is governed by a business agreement (BAA) between HIN and HBI. No PHI was released during the analysis. Instead, HBI implemented a product that was the foundation for the agreement and then reported their results from applying this model to the products/services that HIN now provides. Because this study analyzed de-identified data, the Stanford University Institutional Review Board considered it exempt (October 16, 2014).

### Data warehouse

The data warehouse used in this study consists of all the aggregated patient histories found in Maine’s HIE. Incorporated data elements from the HIE EMRs included patient encounter (visit/admission) history, patient demographics, abnormal laboratory tests and results, radiographic procedures, medication prescriptions, diagnosis and procedures, which were coded according to the *International Classification of Diseases*, *9*^*th*^ *Revision*, *Clinical Modification* (ICD-9-CM), and AHRQ Chronic Condition Indicator (CCI) [25]. Census data from the U.S. Department of Commerce Census Bureau were integrated into our data warehouse. We categorized patients by socioeconomic status utilizing residence zip codes as an approximation to the average household mean and median family income and average level of education. The details of data extraction, management and storage are described in S1 Text.

Maine HIE patient clinical histories were organized as hospital episode level relational database tables. We processed the database at the patient level based on the Enterprise Master Patient Index (EMPI) for population analysis within the HIE’s 36 inpatient and 400+ ambulatory facilities. Since the time point of first confirmatory diagnosis of T2DM varies for each patient, all cohort patients’ first confirmatory diagnoses of T2DM were aligned as time zero. A longitudinal analysis was performed monthly up to 24 months prior to time zero. Multiple patient-level database tables were transformed, integrated, and pivoted to construct a high dimensional table (33,403 clinical variables available for each patient) to support the machine learning process. To reduce the table dimension, i.e. the number of variables for each patient, we exploited the data variance for each data category (primary diagnosis/procedure, secondary diagnosis/procedure, laboratory result, radiology result and outpatient prescription) as the simplest criterion [24], which essentially projects the data points along the dimensions of maximum variances. This dimension reduction exercise ended with a set of 257 clinical longitudinal features from the preceding 24 months before time zero (S1 Table).

### Study design

The overall study design was illustrated in Fig 1. With high throughput genomics datasets, we previous discovered the critical transition point or the early detection of diabetes through the application of the TNE and DDN. In this study, we tested our hypothesis that high dimensional clinical patterns in EMR can be used to define a pre-disease critical transition state prior to definitive T2DM diagnosis. The hypothesis was tested with newly diagnosed patients’ clinical information that was extracted from EMR administrated by the HIE in Maine. An early-warning DDN and the associated clinical parameters were recognized to define the T2DM pre-disease transition state prior to the emerging confirmative diagnosis.

### Cohort construction

**Fig 8:**
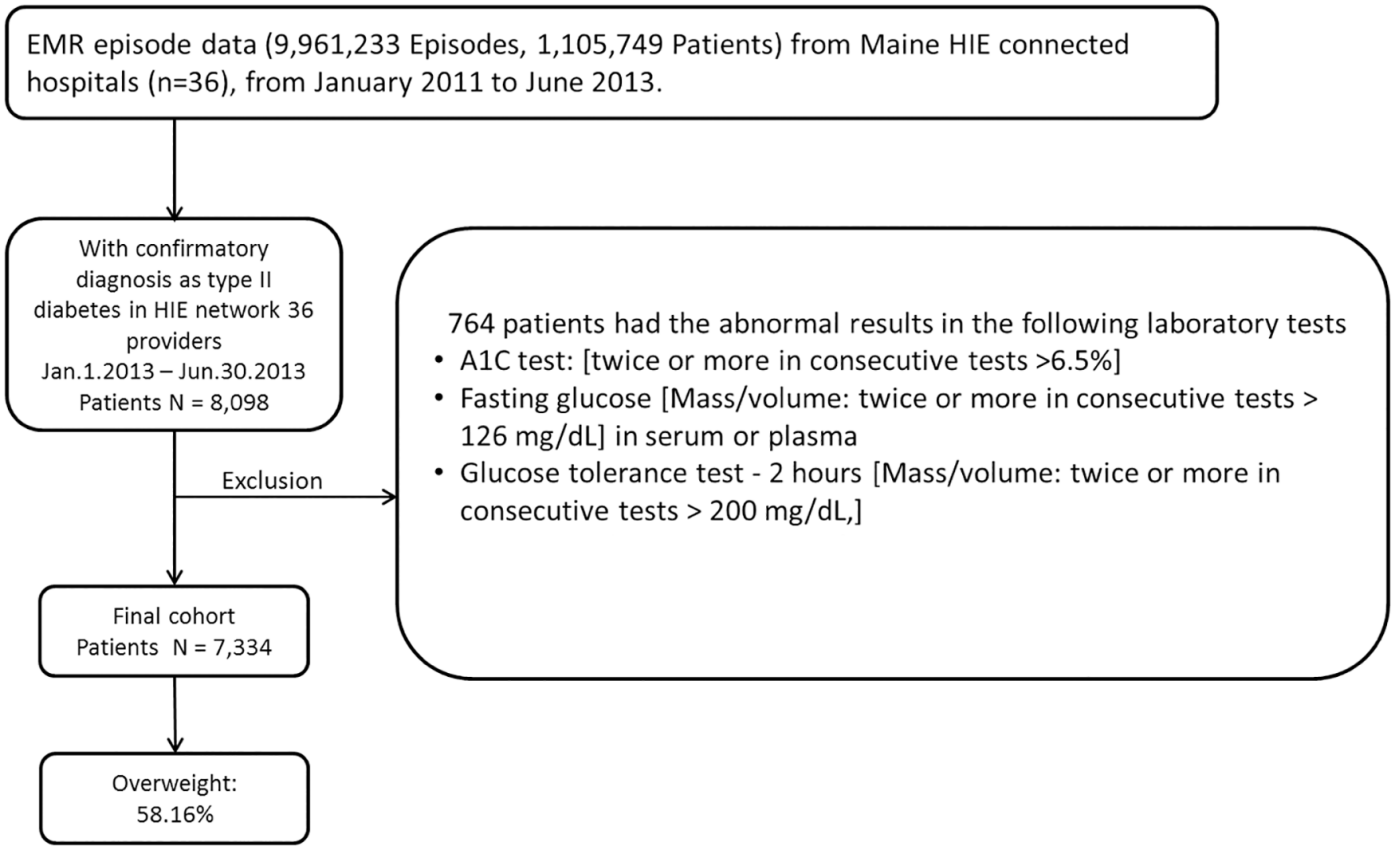
The flow chart of the cohort construction. Based on EMR episode data, a cohort of 8,098 patients was screened out with confirmatory diagnosis as T2DM. 764 patients who had the obviously abnormal results before the confirmative diagnosis in laboratory tests (positive twice) such as fasting glucose test, glucose tolerance test, A1C test, etc., were excluded for analysis, since these subjects might already suffer from T2DM before the confirmative diagnosis. A final cohort of 7,334 patients was constructed. EMR: electronic medical record; HIE: Health information exchange.

Based on the EMR data from 1.3 million people residing in Maine, the ‘case cohort’ considered of those who were alive and diagnosed with T2DM between January 1, 2013 and June 30, 2013. Patients with discharge ICD-9-CM diagnosis codes of 25000, 25002, 25010, 25020, 25022, 25030, 25032, 25040, 25042, 25050, 25052, 25060, 25062, 25070, 25072, 25080, 25082, 25090, and 25092 were considered as having the first confirmatory T2DM diagnosis. Patients who had repeated abnormal glucose laboratory test results of A1C, fasting plasma glucose (FPG), or oral glucose tolerance test (OGTT) before the first confirmatory of T2DM diagnosis were excluded from the case cohort, as they might had developed T2DM before T2DM diagnosis was made by their physicians. For example, the patients who had more than twice the positive results (>6.5%) in A1C test were excluded. The final case cohort included 7,334 patients (Fig 8). Patients without any T2DM-related ICD-9-CM diagnosis codes from June, 2011 to December, 2013 were considered as control. 10,145 age and gender matched controls were selected to construct the control cohort. The demographic data of the case and control cohorts is shown in S2 Table. Compared with control cohort subjects, patients in case cohort exhibited complex comorbidities, making them ‘heavy users’ of healthcare resource with higher costs (p<0.001, Mann—Whitney U test).

### Markov process

Generally, it is difficult to analyze a large network directly because of the complexity and time cost. We used a local network structure instead of the whole network to represent its dynamical properties. Specifically, each node has a local network, i.e., the local network centered on node *i* consists with itself and its *m* linked first-order neighbor nodes *i*_*1*_, *i*_*2*_, …, *i*_*m*_ whose local transition state is *X*^*i*^*(t*) = (x_i_(t), x_i1_(t), …, x_im_(t)) at time *t*.

The transition state coefficient *x*_*i*_ of the Markov process was defined as:

$$x_{i}(t)=\left\{ \begin{array}{l} \begin{array}{ccc} 1 & & if\left|\Delta z_{i}(t)\right|>d_{i} \end{array}\\ \begin{array}{ccc} 0 & & if\left|\Delta z_{i}(t)\right|\leq d_{i} \end{array} \end{array}\right.$$ (1)

Where Δ*z*_*i*_(*t*) = *z*_*i*_(*t*)-*z*_*i*_(*t*-1) is the changing of *z*_*i*_ at time *t*, *d*_*i*_ is the threshold for discretization.

When |Δz_i_(t)| is sufficiently large (> *d*_*i*_), the transition state coefficient *x*_*i*_ = 1, otherwise *x*_*i*_ = 0.

Based on this definition, *X*(*t*) = (*x*_1_(*t*), …, *x*_*n*_(*t*)) is the transition state coefficient for the network at time *t*.

In a local network centered on node *i* with *m* linked first-order neighbors *i*_*1*_, *i*_*2*_, …, *i*_*m*_, we first need to determine the threshold parameters $d=\{d_{i},d_{i_{1}},\cdots ,d_{i_{m}}\}$, which measure whether the state *z*_*i*_(*t*) has a large change or a state transition from its former state *z*_*i*_(*t*-1). Note that a system is in a stable state during its normal state, whereas a system is sensitive to perturbation in its transition state. Thus, *d*_*i*_ should be set to distinguish the “small changes” in normal state from the “large changes” in transition state.

For this local network, therefore, we select $d=\{d_{i},d_{i_{1}},\cdots ,d_{i_{m}}\}$ when the system is in a normal state such that for each node *k*, *p*(|*z*_*k*_ (*t*_0_)| > *d*_*k*_) = *α*, if time point *t*_*0*_ is in a normal state.

Obviously, for such threshold *d*, it holds that

$$p(\left|z_{k}(t_{0})\right|>d_{i},\left|z_{i_{1}}(t_{0})\right|>d_{i_{1}},\cdots ,\left|z_{i_{m}}(t_{0})\right|>d_{i_{m}})\leq \alpha$$ (2)

With such a distribution, features with large deviations clearly correspond to high probabilities.

The following description and derivation are all based on a local node, i.e. *i*. To simplify the notation, we omit *i* and denote *X*^*i*^(*t*) as *X*(*t*), while we also denote the transition state simply as a state.

Given the current state at time *t* is *X*(*t*), at the next time point *t*+1 there are a total of 2^m+1^ possible transition states, each of which is a stochastic event that is denoted, respectively, as ${\left\{A_{u}\right\}}_{u=1,2,\cdots ,2^{m+1}}$, where

$$A_{u}=\{x_{i}=\gamma_{0},x_{i_{1}}=\gamma_{1},\cdots ,x_{i_{m}}=\gamma_{m}\}$$ (3)

where *γ*_*l*_ ∈ {0,1}, for *l* ∈ {0,1,2,…, *m*}. The next state depends on the current state and its transition functions. Obviously, for this local subnetwork, the discrete stochastic process

$${\left\{X(t+i)\right\}}_{i=0,1,\cdots }=\{X(t),X(t+1),\cdots ,X(t+i),\cdots \}$$ (4)

With *X*(*t* + *i*) = *A*_*u*_, *u* ∈ {1,2,…,2^*m*+1^}, is a stochastic Markov process during a period or phase of the system. This stochastic process is defined or given by a Markov matrix *P* = (*p*_*u*,*v*_), which describes the transition rates from state *u* to state *v* as follows

$$p_{u,v}(t)=\text{Pr}(X(t+1))=A_{v}|X(t)=A_{u}$$ (5)

where *u*, *v* ∈ {1,2, …,2^*m*+1^}, and ∑_*v*_ *p*_*u*,*v*_ (*t*) = 1.

### Transition-based network entropy (TNE)

The TNE is a type of Shannon entropy [36] that is conditional on the previous state of the local dynamical network in a Markov process. In the feature space network, each node represents a feature, and each edge represents a regulatory relation between two features. For a local structure centered on node *i* and its *m* linked first-order neighbor nodes *i*_*1*_, *i*_*2*_, …, *i*_*m*_, we already know that its state transition process is a stochastic Markov process. Within a period or phase, we assume that there is no change in the transition matrix, i.e., the transition probabilities *p*_*u*,*v*_ (*t*), between any two possible states *A*_*u*_ and *A*_*v*_. Thus, the process ${\left\{X(t)\right\}}_{t\in [t_{1},t_{2}]}$ is a stationary stochastic Markov process during a specific period [*t*_*1*_, *t*_*2*_], i.e., the normal stage or the transition stage.

Thus, there is a stationary distribution $\pi =(\pi_{1},\cdots ,\pi_{2^{m+1}})$ that satisfies ∑_*v*_ *π*_*v*_*p*_*u*,*v*_ = *π*_*u*_. Therefore, we can define the transition-based network entropy, i.e., the TNE, as

$$H_{i}(t)=H(\chi )=-\sum_{u,v}\pi_{v}p_{u,v}\text{log}p_{u,v}$$ (6)

where the subscript index *i* indicates the center node *i* of this local network, while *χ* represents the transition process *X*(*t*), *X*(*t* + 1), …, *X*(*t* + *T*), … of the local network. The entropy is equivalent to the so-called entropy rate

$$\begin{array}{l} H(\chi )=:\underset{T\to \infty }{\text{lim}}\frac{1}{T}H(X(t),X(t+1),\cdots ,X(t+T))=\underset{T\to \infty }{\text{lim}}H(X(t+T)|\\ X(t+T-1),H(t+T-2),\cdots ,X(t)) \end{array}$$ (7)

when the limit exists. The TNE is the conditional entropy and describes the average transition entropy, depending on the state transition, i.e.*H*_*i*_(*t*) = *H*(*X*(*t*) ∨ *X*(*t*– 1)) = *H*(*X*(*t*), *X*(*t*– 1)–*H*(*X*(*t* − 1))). We also note that *X*(*t*) or (*Z*(*t*)–*Z*(*t*– 1)) are variation variables. Clearly, in a normal state (or a disease state), a system recovers from a small perturbation quickly because of high resilience, i.e., *X*(*t*) and *X*(*t*-1) are almost independent. Thus, we have *H*_*i*_ (*t*) ≈ *H*(*X*(*t*)) due to, *H*(*X*(*t*), *X*(*t*– 1)) ≈ *H*(*X*(*t*)) + *H*(*X*(*t*– 1)) > 0 which results in a high TNE. By contrast, the system has difficulty recovering from a small perturbation in a transition state because of low resilience, i.e., *X*(*t*) and *X*(*t*-1) are strongly correlated, which implies that *H*_*i*_(*t*) rapidly approaches the minimum, *H*_*i*_(*t*) ≈ 0 due to *H*(*X*(*t*), *X*(*t*– 1)) ≈ *H*(*X*(*t*– 1)).

### Dynamic driver network (DDN)

The TNE method described above was employed to identify the DDN. Considering the following equations with noise perturbations near the equilibrium $\bar{Z}$:

Z(t+1)=f(Z(t);P) (8)

Where *Z*(*t*) = (*z*_1_(*t*), …, *z*_*n*_(*t*)) is an *n*-dimensional state vector or variables at time *t* that represent features in the system, while *P* = (*p*_*1*_,…, *p*_*s*_) is a parameter vector or driving factors that represent slowly changing factors. Mappings *f*: *R*^*n*^ × *R*^*s*^ → *R*^*n*^ are generally nonlinear functions. For such dynamical evolution function, there is a bifurcation if the following conditions hold:

1. $\bar{Z}$ is a fixed point of system such that $\bar{Z}=f(\bar{Z};P)$.
2. There is a value *P*_*c*_ such that one or a pair of the eigenvalues of the Jacobian matrix $\frac{\partial f(Z;P_{C})}{\partial Z}{|}_{Z=\bar{Z}}$ is equal to 1 in the modulus.
3. When *P* ≠ *P*_*c*_, the eigenvalues are not always equal to 1 in the modulus.

The above three conditions with other transverse conditions imply that the system undergoes a phase change at $\bar{Z}$ or a codimension-one bifurcation when *P* reaches the threshold *P*_*c*_. For system near $\bar{Z}$ and before *P* reaches *P*_*c*_, we assume that the system is at a stable fixed point $\bar{Z}$ so all the eigenvalues are within (0, 1) in the modulus. The parameter value *P*_*c*_ at which the state shift of the system occurs, is known as a bifurcation parameter value or a critical transition value.

The generic properties in dynamics of Eq (8) was derived based on a consideration of the linearized equations for Eq (8) and the noise perturbations near $\bar{Z}$. Specifically, introducing new variables *Y*(*t*) = (*Y*_1_(*t*), …,*Y*_*n*_(*t*)) and a transformation matrix *S*, i.e., $Y(t)=S^{-1}(Z(t)-\bar{Z})$, we have

Y(t+1)=Λ(P)Y(t)+ς(t) (9)

where Λ(*P*) is the diagnosis matrix of $\frac{\partial f(Z;P_{C})}{\partial Z}{|}_{Z=\bar{Z}}$, *ς*(*t*) = (*ς*_1_(*t*), …, *ς*_*n*_(*t*)) are small Gaussian noises with zero means.

We denote *σ*_*i*_ as the small standard deviation of *ζ*_*i*_ for all *k*. Without any loss of generality, the diagnosis matrix Λ = (*λ*_*1*_,…, *λ*_*n*_) for each *λ*_*i*_ is between 0 and 1. (In fact, three typical cases arise during the diagonalization process [15], but we only illustrate the diagonal case with different real eigenvalues for simplicity. The derivations of the other two cases are similar. Among all eigenvalues of *Λ*, the largest one (in the modulus), say λ_1_, approaches 1 in the modulus when parameter *P* →*P*_*c*_. The eigenvalue *λ*_*1*_ characterizes the system's rate of change around a fixed point and is known as the dominant eigenvalue. The normal state corresponds to a period of |*λ*_1_| < 1, whereas the transition stage corresponds to the period with *λ*_1_ → 1. Without generality loss, we assume that the first variable *y*_1_ in *Y* corresponds to *λ*_1_, namely (*y*_1_, 0, …, 0) is the eigenvector of *λ*_1_. Close to a fixed point, we have shown that there is a dominant group or a dynamical driver network (DDN) that satisfies the following conditions when the system approaches a critical transition point [15].

We now summarize the critical properties of the stochastically perturbed linear system as described in Eq (9). Denote *z*_*i*_ as the value of feature *i*. When *P* approaches the bifurcation point, the following results hold:

1. For any feature *i* in the DDN, the fluctuation of *z*_*i*_ increase sharply;
2. If both *i* and *j* are in the DDN, then the correlation between *z*_*i*_ and *z*_*j*_ sharply increases;
3. If *i* is in the DDN but *j* is not, then the correlation between *z*_*i*_ and *z*_*j*_ decrease sharply;
4. For two features *i* nor *j* which do not belong to the DDN, the correlation between *z*_*i*_ and *z*_*j*_ show no significant changes.

This result describes the generic critical properties of features in DDN. The DDN is the sub-network that makes the first move from one state toward another state at the critical transition point, so we refer to the DDN as the driving network in this critical transition.

In contrast to the original variables *Z* analysis [15], this work focuses on the variation equation for Eq (8) with variation variables Δ*Z*. We note that

$$z_{i}=s_{ij}y_{1}+\cdots +s_{in}y_{n}+{\bar{z}}_{l}$$ (10)

Let the variation variables be Δ*Z* = *Z*(*t*)-*Z*(*t*-1), then Eq (10) is transformed into

$$\Delta z_{i}=s_{ij}\Delta y_{1}+\cdots +s_{in}\Delta y_{n}$$ (11)

Where

ΔY=Y(t)−Y(t−1) (12)

We refer to Δ*z*_*i*_(*t*) and Δ*y*_*i*_(*t*) as the variation variables for *z*_*i*_(*t*) and *y*_*i*_(*t*), respectively. From Eqs (9) and (12), it clearly holds that

ΔY(t+1)=ΔY(t)+ξ(t) (13)

where *ξ*(*t*) = *ς*(*t*) − *ς*(*t* − 1) are Gaussian noises with zero means and covariances *k*_*ij*_ = *Cov*(*ξ*_*i*_, *ξ*_*j*_). It is clear that the standard deviation of *ξ*_*i*_(*t*) is $\sqrt{2}\sigma_{i}$, where *σ*_*i*_ is the standard deviation of *ζ* for all *t*. The variable *Δy*_*1*_ corresponds to the dominant eigenvalue *λ*_*1*_.

For a stochastically perturbed linear system of Eq (8):

Z(t+1)=A(P)Z(t)+ε(t) (14)

where *ε*(*t*) is the Gaussian noise and *P* is a parameter vector that controls the Jacobian matrix *A*. We denote the variation variable as Δ*z*_*i*_(*t*) = *z*_*i*_(*t*)—*z*_*i*_(*t-1*) and assume *T* to be sufficiently large.

1. When *P* is not in the vicinity of a critical transition point or a bifurcation point, it holds that for any *i* and *j* including *i* = *j*, Δ*z*_*i*_(*t*+*T*) is statistically independent of Δ*z*_*j*_(*t*), where *i*, *j* = 1,2,…,*n*.
2. When *P* approaches to a critical transition point, the following holds.
  - If both *i* and *j* are in the dominant group, or DDN members, then there is a strong correlation between Δ*z*_*i*_(*t*+*T*) and Δ*z*_*j*_(*t*);
  - If neither *i* nor *j* in the dominant group, then Δ*z*_*i*_(*t*+*T*) is statistically independent of Δ*z*_*j*_(*t*). Note that this also holds for *i* = *j*.

We point out that for a nonlinear case at a fixed point, the dynamical behavior has the same trend described.

We combine the TNEs for all nodes and define the average network entropy for the whole network with *n* nodes as the average TNE as follows:

$$H(t)=\frac{1}{n}\sum_{i=1}^{n}H_{i}(t)$$ (15)

Suppose that there are control samples and case samples, then we define the comparative entropy as:

$$E(t)=\frac{H_{control}(t)}{H_{case}(t)}$$ (16)

where *H*_*control*_(*t*) is the TNE based on control samples in the form of Eq (15), and *H*_*case*_(*t*) is the TNE based on case samples in the form of Eq (15).

## Supporting information

S1 Fig The dynamical progression of feature networks of case and control population. (A). The dynamical progression of feature networks of case population; (B). The dynamical progression of feature networks of control population.
(TIFF)

S1 Text Data extraction, management and storage. (DOCX)

S1 Table A list of DDN features. (DOCX)

S2 Table The demographic characteristics of the case and control cohort. (DOCX)
