# Supplementary figures and images for: Defining and characterizing the critical transition state prior to the type 2 diabetes disease
Source: PLoS One. 2017 Jul 7;12(7):e0180937. doi: 10.1371/journal.pone.0180937 (PMC5501620; doi:10.1371/journal.pone.0180937)

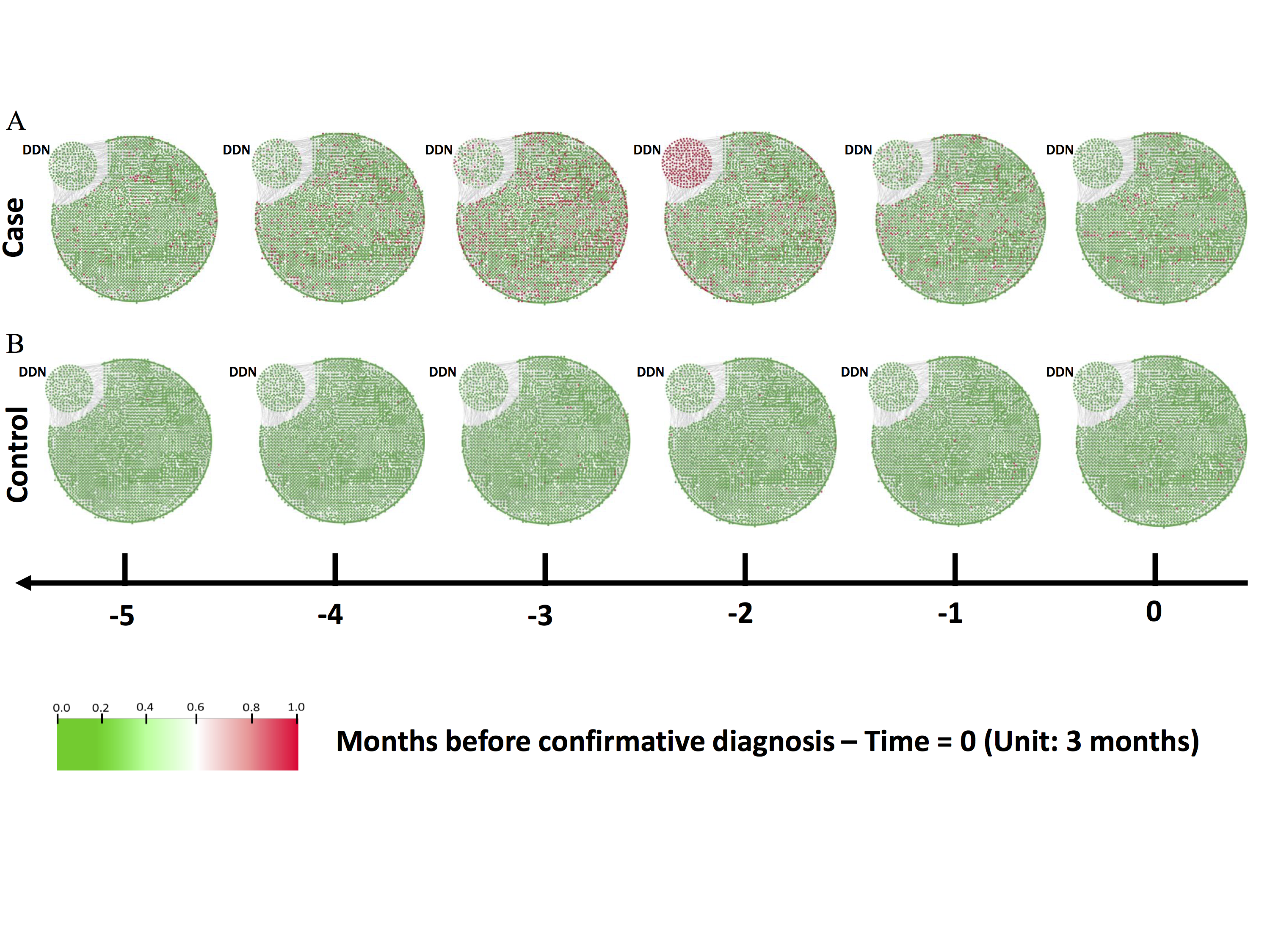

Supplement: S1 Fig — (A). The dynamical progression of feature networks of case population; (B). The dynamical progression of feature networks of control population. (TIFF) [file pone.0180937.s001.tiff]
