# Supplementary material for: Defining and characterizing the critical transition state prior to the type 2 diabetes disease
Source: PLoS One. 2017 Jul 7;12(7):e0180937. doi: 10.1371/journal.pone.0180937 (PMC5501620; doi:10.1371/journal.pone.0180937)
Supplement: S1 Text — (DOCX) [file pone.0180937.s002.docx]

**S1 Text. Data extraction, management and storage**

**Maine Health Information Exchange (HIE) is connected to the majority of health care facilities in Maine, including 376 physician offices, 12 behavioral health facilities, 15 critical access hospitals, 37 federally qualified health centers (FQHC), 23 hospitals, and 12 long-term care facilities. HIE includes records for 1.3 million individuals including in-state and out-of-state residents. Over 90% of Maine residents have a record in the database. HealthInfoNet is an independent, nonprofit organization operating the HIE in Maine. It maintains an opt-out consent process for general medical information, and an opt-in patient consent for certain behavioral health and HIV related information, as required by Maine State law. The HIE has just over a 1% patient opt-out rate. Incorporated data elements include patient demographic information, laboratory tests and results, radiographic procedures (Logical Observation Identifiers Names and Codes), medication prescriptions (RxNorm), diagnosis and procedures which are coded according to the International Classification of Diseases, 9th/10th Revision, Clinical Modification (ICD-9-CM/ICD-10-CM). Census data from the U.S. Department of Commerce Census Bureau were integrated into our data warehouse, to provide approximation on patients’ socioeconomic status information in terms of the average household mean and median family income and average degree of educational attainment, based on residence zip codes.**

**An enterprise standard business process is implemented on Orion Health Rhapsody integration engine by HIE to ensure robust and reliable acquisition and exchange of high volume EMR health data. HBI Solutions, Inc. managed a sequential staging data warehouse to extract, transform and load entire EMR data from HIE system. Data cleaning and integration were applied for handling error and data quality issue. And then an analysis database including data attribute described in method part was built based on the staging database for learning and prediction process.**

**The work was performed under a business and development arrangement between HealthInfoNet (http://www.hinfonet.org), the operators of the Maine HIE and HBI located in California. HBI is a subcontractor to HealthInfoNet to develop and implement predictive risk models to be used by HealthInfoNet member providers. HealthInfoNet is responsible for security and access to its members' data and has established data service agreements (DSAs) restricting unnecessary exposure of information. All data analysis and modeling for this study was performed on HealthInfoNet servers, and data was accessed via secure connections controlled by HealthInfoNet.**
