# Supplementary material for: Defining and characterizing the critical transition state prior to the type 2 diabetes disease
Source: PLoS One. 2017 Jul 7;12(7):e0180937. doi: 10.1371/journal.pone.0180937 (PMC5501620; doi:10.1371/journal.pone.0180937)
Supplement: S1 Table — (DOCX) [file pone.0180937.s003.docx]

**S1 Table. A list of DDN features**

| **Category** | **Name** | **Function** | **Disease** | **Target** |
| --- | --- | --- | --- | --- |
| Chronic Disease | Anxiety disorders |  |  |  |
| Abnormal Laboratory Test | Basic metabolic 2000 panel in Serum or Plasma-Abnormal |  |  |  |
| Abnormal Laboratory Test | Cardiac Panel-Abnormal |  |  |  |
| Abnormal Laboratory Test | CBC with Ordered Manual Differential panel in Blood-Abnormal |  |  |  |
| Abnormal Laboratory Test | Complete blood count (hemogram) panel in Blood by Automated count-Abnormal |  |  |  |
| Abnormal Laboratory Test | Comprehensive metabolic 2000 panel in Serum or Plasma-Abnormal |  |  |  |
| Abnormal Laboratory Test | INR in Capillary blood by Coagulation assay-Abnormal |  |  |  |
| Abnormal Laboratory Test | Urinalysis complete W Reflex Culture panel in Urine-Abnormal |  |  |  |
| Abnormal Laboratory Test | Urinalysis dipstick W Reflex Microscopic panel in Urine-Abnormal |  |  |  |
| Medication Prescriptions | ADVAIR 500-50 DISKUS (combinations of fluticasone propionate and salmeterol xinafoate) | Fluticasone propionate is a synthetic trifluorinated corticosteroid; Salmeterol is a long-acting selective beta2-adrenergic agonist | Asthma | Fluticasone propionate is a synthetic trifluorinated corticosteroid with potent anti-inflammatory activity; Salmeterol is a long-acting beta2-adrenergic agonist |
| Medication Prescriptions | AFLURIA 2012-2013 SYRINGE(Influenza Vaccine) | inactivated influenza vaccine | Flu | indicated for active immunization against influenza disease caused by influenza virus subtypes A and type B present in the vaccine |
| Medication Prescriptions | ALLOPURINOL TAB 100MG | a xanthine oxidase inhibitor | gout; recurrent calcium oxalate calculi; leukemia, lymphoma and malignancies who are receiving cancer therapy which causes elevations of serum and urinary uric acid levels | reduces serum and urinary uric acid concentrations |
| Medication Prescriptions | ALLOPURINOL 100 MG TABLET | a xanthine oxidase inhibitor | gout; recurrent calcium oxalate calculi; leukemia, lymphoma and malignancies who are receiving cancer therapy which causes elevations of serum and urinary uric acid levels | reduces serum and urinary uric acid concentrations |
| Medication Prescriptions | AMIODARONE TAB 200MG | treatment and prophylaxis of frequently recurring ventricular fibrillation and hemodynamically unstable ventricular tachycardia in patients refractory to other therapy | ventricular arrhythmias | works directly on the heart tissue and will slow the nerve impulses in the heart |
| Medication Prescriptions | AMIODARONE HCL 200 MG TABLET | treatment and prophylaxis of frequently recurring ventricular fibrillation and hemodynamically unstable ventricular tachycardia in patients refractory to other therapy | ventricular arrhythmias | works directly on the heart tissue and will slow the nerve impulses in the heart |
| Medication Prescriptions | AMITRIPTYLIN TAB 10MG | antidepressant with sedative effects | symptoms of depression | inhibits the membrane pump mechanism responsible for uptake of norepinephrine and serotonin in adrenergic and serotonergic neurons |
| Medication Prescriptions | AMITRIPTYLINE HCL 10 MG TAB | antidepressant with sedative effects | symptoms of depression | inhibits the membrane pump mechanism responsible for uptake of norepinephrine and serotonin in adrenergic and serotonergic neurons |
| Medication Prescriptions | AMITRIPTYLINE HCL 50 MG TAB | antidepressant with sedative effects | symptoms of depression | inhibits the membrane pump mechanism responsible for uptake of norepinephrine and serotonin in adrenergic and serotonergic neurons |
| Medication Prescriptions | AMLODIPINE TAB 5MG | a long-acting calcium channel blocker | Hypertension; Coronary Artery Disease | relaxes (widens) blood vessels and improves blood flow |
| Medication Prescriptions | AMLODIPINE BESYLATE 2.5 MG TAB | a long-acting calcium channel blocker | Hypertension; Coronary Artery Disease | relaxes (widens) blood vessels and improves blood flow |
| Medication Prescriptions | AMOXICILLIN CAP 500MG | a semisynthetic antibiotic, an analog of ampicillin, with a broad spectrum of bactericidal activity against many Gram-positive and Gram-negative microorganisms | Infections of the ear, nose, and throat; Infections of the genitourinary tract; Infections of the skin and skin structure; Infections of the lower respiratory tract; Gonorrhea, acute uncomplicated ;Triple therapy for Helicobacter pylori with clarithromycin and lansoprazole; | stop bacteria from multiplying by preventing bacteria from forming the walls that surround them |
| Medication Prescriptions | AMOXICILLIN 500 MG CAPSULE | a semisynthetic antibiotic, an analog of ampicillin, with a broad spectrum of bactericidal activity against many Gram-positive and Gram-negative microorganisms | Infections of the ear, nose, and throat; Infections of the genitourinary tract; Infections of the skin and skin structure; Infections of the lower respiratory tract; Gonorrhea, acute uncomplicated ;Triple therapy for Helicobacter pylori with clarithromycin and lansoprazole; | stop bacteria from multiplying by preventing bacteria from forming the walls that surround them |
| Medication Prescriptions | ASACOL TAB 400MG DR | anti-inflammatory drug | moderately active ulcerative colitis and maintenance of remission of ulcerative colitis | works inside the intestines (bowel) to reduce the inflammation and other symptoms of the disease |
| Medication Prescriptions | ASACOL EC 400 MG TABLET | anti-inflammatory drug | moderately active ulcerative colitis and maintenance of remission of ulcerative colitis | works inside the intestines (bowel) to reduce the inflammation and other symptoms of the disease |
| Medication Prescriptions | ASPIRIN EC 81 MG TABLET | nonsteroidal anti-inflammatory drug | prevent heart attack or stroke | temporarily relieves minor aches and pains; prevents blood from clotting |
| Medication Prescriptions | ATENOLOL TAB 25MG | treatment of hypertension | Hypertension; Angina Pectoris Due to Coronary Atherosclerosis; Acute Myocardial Infarction | a beta1-selective (cardioselective) hydrophilic blocking agent |
| Medication Prescriptions | ATORVASTATIN | lowers the level of cholesterol in the blood | prevent certain types of heart and blood vessel problems in patients with risk factors for heart problems | inhibitor of 3-hydroxy-3-methylglutaryl-coenzyme A (HMG-CoA) reductase . This enzyme catalyzes the conversion of HMG-CoA to mevalonate, an early and rate-limiting step in cholesterol biosynthesis. |
| Medication Prescriptions | ATORVASTATIN 10 MG TABLET | lowers the level of cholesterol in the blood | prevent certain types of heart and blood vessel problems in patients with risk factors for heart problems | inhibitor of 3-hydroxy-4-methylglutaryl-coenzyme A (HMG-CoA) reductase . This enzyme catalyzes the conversion of HMG-CoA to mevalonate, an early and rate-limiting step in cholesterol biosynthesis. |
| Medication Prescriptions | ATORVASTATIN 80 MG TABLET | lowers the level of cholesterol in the blood | prevent certain types of heart and blood vessel problems in patients with risk factors for heart problems | inhibitor of 3-hydroxy-5-methylglutaryl-coenzyme A (HMG-CoA) reductase . This enzyme catalyzes the conversion of HMG-CoA to mevalonate, an early and rate-limiting step in cholesterol biosynthesis. |
| Medication Prescriptions | ATORVASTATIN TAB 10MG | lowers the level of cholesterol in the blood | prevent certain types of heart and blood vessel problems in patients with risk factors for heart problems | inhibitor of 3-hydroxy-6-methylglutaryl-coenzyme A (HMG-CoA) reductase . This enzyme catalyzes the conversion of HMG-CoA to mevalonate, an early and rate-limiting step in cholesterol biosynthesis. |
| Medication Prescriptions | ATORVASTATIN TAB 40MG | lowers the level of cholesterol in the blood | prevent certain types of heart and blood vessel problems in patients with risk factors for heart problems | inhibitor of 3-hydroxy-7-methylglutaryl-coenzyme A (HMG-CoA) reductase . This enzyme catalyzes the conversion of HMG-CoA to mevalonate, an early and rate-limiting step in cholesterol biosynthesis. |
| Medication Prescriptions | AVODART 0.5 MG SOFTGEL | prevents the conversion of testosterone to dihydrotestosterone (DHT) in the body | symptomatic benign prostatic hyperplasia (BPH) | a selective inhibitor of both the type 1 and type 2 isoforms of steroid 5 alpha-reductase, an intracellular enzyme that converts testosterone to DHT |
| Medication Prescriptions | AZITHROMYCIN 250 MG TABLET | macrolide antibiotics | Acute bacterial exacerbations of chronic obstructive pulmonary disease; Acute bacterial sinusitis; Community-acquired pneumonia | killing bacteria or preventing their growth |
| Medication Prescriptions | AZITHROMYCIN TAB 250MG | macrolide antibiotics | Acute bacterial exacerbations of chronic obstructive pulmonary disease; Acute bacterial sinusitis; Community-acquired pneumonia | killing bacteria or preventing their growth |
| Medication Prescriptions | BACLOFEN 10 MG TABLET | muscle relaxant and antispastic;the alleviation of signs and symptoms of spasticity resulting from multiple sclerosis | alcoholism; multiple sclerosis,particularly for the relief of flexor spasms and concomitant pain, clonus, and muscular rigidity | an agonist for the GABAB receptors |
| Medication Prescriptions | VESICARE (solifenacin succinate) TAB 10MG | muscarinic receptor antagonist | overactive bladder with symptoms of urge urinary incontinence, urgency, and urinary frequency | muscles of the bladder to prevent them from causing incontinence |
| Medication Prescriptions | VITAMIN D 1,000 UNIT TABLET | growth of teeth and bones |  |  |
| Medication Prescriptions | WARFARIN | an anticoagulant | venous thrombosis; pulmonary embolism; atrial fibrillation ; myocardial infarction | acts by inhibiting vitamin K-dependent coagulation factors |
| Medication Prescriptions | WARFARIN TAB 1MG | an anticoagulant | venous thrombosis; pulmonary embolism; atrial fibrillation ; myocardial infarction | acts by inhibiting vitamin K-dependent coagulation factors |
| Medication Prescriptions | WARFARIN TAB 5MG | an anticoagulant | venous thrombosis; pulmonary embolism; atrial fibrillation ; myocardial infarction | acts by inhibiting vitamin K-dependent coagulation factors |
| Medication Prescriptions | WARFARIN SODIUM 5 MG TABLET | an anticoagulant | venous thrombosis; pulmonary embolism; atrial fibrillation ; myocardial infarction | acts by inhibiting vitamin K-dependent coagulation factors |
| Medication Prescriptions | ZETIA TAB 10MG | in a class of lipid-lowering compounds | atherosclerotic vascular disease, Primary Hyperlipidemia | selectively inhibits the intestinal absorption of cholesterol and related phytosterols |
| Medication Prescriptions | ZETIA 10 MG TABLET | in a class of lipid-lowering compounds | atherosclerotic vascular disease, Primary Hyperlipidemia | selectively inhibits the intestinal absorption of cholesterol and related phytosterols |
| Medication Prescriptions | ZOLPIDEM | a gamma-aminobutyric acid (GABA) A agonist | short-term treatment of insomnia | decrease sleep latency |
| Medication Prescriptions | ZOLPIDEM TAB 5MG | a gamma-aminobutyric acid (GABA) A agonist | short-term treatment of insomnia | decrease sleep latency |
| Medication Prescriptions | ZOLPIDEM TARTRATE 5 MG TABLET | a gamma-aminobutyric acid (GABA) A agonist | short-term treatment of insomnia | decrease sleep latency |
| Medication Prescriptions | ZOSTAVAX VIAL(attenuated varicella-zoster virus ) | prevention of herpes zoster |  |  |
| Medication Prescriptions | BUMETANIDE 1 MG TABLET | loop diuretics ,treat fluid retention (edema) and swelling | congestive heart failure, liver disease, kidney disease, or other medical conditions | It works by acting on the kidneys to increase the flow of urine . |
| Medication Prescriptions | BUPROPION HCL XL 300 MG TABLET | antidepressant medication | depression, other mental/mood disorders, and smoking cessation | chemicals within the brain that nerves use to send messages to each other |
| Medication Prescriptions | BUSPIRONE | antianxiety agent | anxiety disorders or the short-term relief of the symptoms of anxiety | unknown, not chemically or pharmacologically related to the benzodiazepines, barbiturates, or other sedative/anxiolytic drugs; maybe have a high affinity for serotonin (5-HT1A) receptors |
| Medication Prescriptions | CALCITRIOL CAP 0.5MCG | a synthetic vitamin D analog which is active in the regulation of the absorption of calcium from the gastrointestinal tract and its utilization in the body | Predialysis,Dialysis,Hypoparathyroidism | Calcitriol, a pharmaceutical form of vitamin D, has anti-osteoporotic, immunomodulatory, anticarcinogenic, antipsoriatic, antioxidant, and mood-modulatory activities |
| Medication Prescriptions | CALCITRIOL 0.25 MCG CAPSULE | a synthetic vitamin D analog which is active in the regulation of the absorption of calcium from the gastrointestinal tract and its utilization in the body | Predialysis,Dialysis,Hypoparathyroidism | Calcitriol, a pharmaceutical form of vitamin D, has anti-osteoporotic, immunomodulatory, anticarcinogenic, antipsoriatic, antioxidant, and mood-modulatory activities |
| Medication Prescriptions | CARBAMAZEPINE 200 MG TABLET | an anticonvulsant and specific analgesic for trigeminal neuralgia | Epilepsy,Trigeminal Neuralgia | works in the brain and nervous system to control seizures, pain, and bipolar disorder |
| Medication Prescriptions | CARBIDOPA-LEVODOPA 25-100 TAB | combination of carbidopa and levodopa for the treatment of Parkinson's disease and syndrome | the symptoms of idiopathic Parkinson's disease (paralysis agitans), post-encephalitic parkinsonism, and symptomatic parkinsonism which may follow injury to the nervous system by carbon monoxide intoxication and/or manganese intoxication | Carbidopa, an inhibitor of aromatic amino acid decarboxylation; Levodopa, an aromatic amino acid |
| Medication Prescriptions | CARVEDILOL TAB 3.125MG | reduces the heart's rate and force of contraction and thereby reduces the work of the heart; causes the arteries to relax and the blood pressure to fall | Heart Failure; Left Ventricular Dysfunction Following Myocardial Infarction; Hypertension | Carvedilol is a nonselective β-adrenergic blocking agent with α1-blocking activity |
| Medication Prescriptions | CARVEDILOL 25 MG TABLET | reduces the heart's rate and force of contraction and thereby reduces the work of the heart; causes the arteries to relax and the blood pressure to fall | Heart Failure; Left Ventricular Dysfunction Following Myocardial Infarction; Hypertension | Carvedilol is a nonselective β-adrenergic blocking agent with α2-blocking activity |
| Medication Prescriptions | CEPHALEXIN | a semisynthetic cephalosporin antibiotic intended for oral administration | Respiratory tract infections; Otitis media ; Skin and skin structure infections ; Bone infections; Genitourinary tract infections | killing bacteria or preventing their growth |
| Medication Prescriptions | CEPHALEXIN CAP 500MG | a semisynthetic cephalosporin antibiotic intended for oral administration | Respiratory tract infections; Otitis media ; Skin and skin structure infections ; Bone infections; Genitourinary tract infections | killing bacteria or preventing their growth |
| Medication Prescriptions | CIALIS(tadalafil) 20 MG TABLET | an oral treatment for erectile dysfunction | erectile dysfunction | is a selective inhibitor of cyclic guanosine monophosphate (cGMP)-specific phosphodiesterase type 5 (PDE5) |
| Medication Prescriptions | CITALOPRAM TAB 10MG | antidepressant agents | depression | selective serotonin reuptake inhibitor (SSRI), work by increasing the activity of a chemical called serotonin in the brain |
| Medication Prescriptions | CITALOPRAM TAB 20MG | antidepressant agents | depression | selective serotonin reuptake inhibitor (SSRI), work by increasing the activity of a chemical called serotonin in the brain |
| Medication Prescriptions | CITALOPRAM TAB 40MG | antidepressant agents | depression | selective serotonin reuptake inhibitor (SSRI), work by increasing the activity of a chemical called serotonin in the brain |
| Medication Prescriptions | CITALOPRAM HBR 40 MG TABLET | antidepressant agents | depression | selective serotonin reuptake inhibitor (SSRI), work by increasing the activity of a chemical called serotonin in the brain |
| Medication Prescriptions | CLONAZEPAM | benzodiazepine, treatment of anxiety and seizure disorders | Seizure Disorders; Panic Disorder | enhance the activity of gamma aminobutyric acid (GABA), the major inhibitory neurotransmitter in the central nervous system |
| Medication Prescriptions | CLONAZEPAM 1 MG TABLET | benzodiazepine, treatment of anxiety and seizure disorders | Seizure Disorders; Panic Disorder | enhance the activity of gamma aminobutyric acid (GABA), the major inhibitory neurotransmitter in the central nervous system |
| Medication Prescriptions | CLONIDINE HCL 0.1 MG TABLET | antihypertensive agents | hypertension | a centrally acting alpha-agonist hypotensive agent |
| Medication Prescriptions | CLONIDINE HCL 0.2 MG TABLET | antihypertensive agents | hypertension | a centrally acting alpha-agonist hypotensive agent |
| Medication Prescriptions | CLOPIDOGREL | inhibits platelet aggregation | Acute Coronary Syndrome (ACS); Recent MI, Recent Stroke, Or Established Peripheral Arterial Disease | a thienopyridine class inhibitor of P2Y12 ADP platelet receptors |
| Medication Prescriptions | CLOPIDOGREL TAB 75MG | inhibits platelet aggregation | Acute Coronary Syndrome (ACS); Recent MI, Recent Stroke, Or Established Peripheral Arterial Disease | a thienopyridine class inhibitor of P2Y13 ADP platelet receptors |
| Medication Prescriptions | CLOPIDOGREL 75 MG TABLET | inhibits platelet aggregation | Acute Coronary Syndrome (ACS); Recent MI, Recent Stroke, Or Established Peripheral Arterial Disease | a thienopyridine class inhibitor of P2Y14 ADP platelet receptors |
| Medication Prescriptions | COLCRYS 0.6 MG TABLET | prophylaxis and the treatment of acute gout flares | Gout Flares; Prophylaxis of Gout Flares; Treatment of Gout Flares; Familial Mediterranean fever (FMF) | interfere with the intracellular assembly of the inflammasome complex present in neutrophils and monocytes that mediates activation of interleukin-1β. |
| Medication Prescriptions | CONTOUR TEST STRIPS | Blood sugar monitoring |  |  |
| Medication Prescriptions | COUMADIN (Warfarin) TAB 5MG | an anticoagulant | venous thrombosis; pulmonary embolism; atrial fibrillation ; myocardial infarction | acts by inhibiting vitamin K-dependent coagulation factors |
| Medication Prescriptions | CRESTOR TAB 40MG | slows the production of cholesterol | Hyperlipidemia and Mixed Dyslipidemia; Pediatric Patients 10 to 17 years of age with Heterozygous Familial Hypercholesterolemia (HeFH); Hypertriglyceridemia; Primary Dysbetalipoproteinemia (Type III Hyperlipoproteinemia); Homozygous Familial Hypercholesterolemia; Primary Prevention of Cardiovascular Disease | CRESTOR is a selective and competitive inhibitor of HMG-CoA reductase, the rate-limiting enzyme that converts 3-hydroxy-3- methylglutaryl coenzyme A to mevalonate, a precursor of cholesterol |
| Medication Prescriptions | CRESTOR TAB 20MG | slows the production of cholesterol | Hyperlipidemia and Mixed Dyslipidemia; Pediatric Patients 10 to 18 years of age with Heterozygous Familial Hypercholesterolemia (HeFH); Hypertriglyceridemia; Primary Dysbetalipoproteinemia (Type III Hyperlipoproteinemia); Homozygous Familial Hypercholeste | CRESTOR is a selective and competitive inhibitor of HMG-CoA reductase, the rate-limiting enzyme that converts 3-hydroxy-4- methylglutaryl coenzyme A to mevalonate, a precursor of cholesterol |
| Medication Prescriptions | CRESTOR 10 MG TABLET | slows the production of cholesterol | Hyperlipidemia and Mixed Dyslipidemia; Pediatric Patients 10 to 19 years of age with Heterozygous Familial Hypercholesterolemia (HeFH); Hypertriglyceridemia; Primary Dysbetalipoproteinemia (Type III Hyperlipoproteinemia); Homozygous Familial Hypercholeste | CRESTOR is a selective and competitive inhibitor of HMG-CoA reductase, the rate-limiting enzyme that converts 3-hydroxy-5- methylglutaryl coenzyme A to mevalonate, a precursor of cholesterol |
| Medication Prescriptions | CRESTOR 20 MG TABLET | slows the production of cholesterol | Hyperlipidemia and Mixed Dyslipidemia; Pediatric Patients 10 to 20 years of age with Heterozygous Familial Hypercholesterolemia (HeFH); Hypertriglyceridemia; Primary Dysbetalipoproteinemia (Type III Hyperlipoproteinemia); Homozygous Familial Hypercholeste | CRESTOR is a selective and competitive inhibitor of HMG-CoA reductase, the rate-limiting enzyme that converts 3-hydroxy-6- methylglutaryl coenzyme A to mevalonate, a precursor of cholesterol |
| Medication Prescriptions | CYCLOBENZAPR TAB 5MG | relief of muscle spasm associated with acute, painful musculoskeletal conditions | muscle spasm | acts on the central nervous system (CNS) to produce its muscle relaxant effects |
| Medication Prescriptions | CYMBALTA 60 MG CAPSULE | used to treat depression and anxiety. also used for pain caused by nerve damage associated with diabetes | Major Depressive Disorder; Generalized Anxiety Disorder; Diabetic Peripheral Neuropathic Pain; Fibromyalgia; Chronic Musculoskeletal Pain | is selective serotonin and norepinephrine reuptake inhibitors (SSNRIs). work by increasing the activity of chemicals called serotonin and norepinephrine in the brain |
| Medication Prescriptions | DEXILANT CAP 60MG DR | treat certain conditions in which there is too much acid in the stomach | erosive esophagitis ; "heartburn" caused by gastroesophageal reflux disease (GERD) | is a proton pump inhibitor (PPI). works by decreasing the amount of acid produced by the stomach |
| Medication Prescriptions | DICYCLOMINE CAP 10MG | an antispasmodic and anticholinergic | functional bowel/irritable bowel syndrome | relieves smooth muscle spasm of the gastrointestinal tract |
| Medication Prescriptions | DICYCLOMINE 10 MG CAPSULE | an antispasmodic and anticholinergic | functional bowel/irritable bowel syndrome | relieves smooth muscle spasm of the gastrointestinal tract |
| Medication Prescriptions | DIGOXIN TAB 0.125MG | improve the strength and efficiency of the heart, or to control the rate and rhythm of the heartbeat | Heart Failure in Adults; Heart Failure in Pediatric Patients; Atrial Fibrillation in Adults | cardiac (or digitalis) glycosides, effects on the myocardium |
| Medication Prescriptions | DIGOXIN TAB 0.25MG | improve the strength and efficiency of the heart, or to control the rate and rhythm of the heartbeat | Heart Failure in Adults; Heart Failure in Pediatric Patients; Atrial Fibrillation in Adults | cardiac (or digitalis) glycosides, effects on the myocardium |
| Medication Prescriptions | DIGOXIN 125 MCG TABLET | improve the strength and efficiency of the heart, or to control the rate and rhythm of the heartbeat | Heart Failure in Adults; Heart Failure in Pediatric Patients; Atrial Fibrillation in Adults | cardiac (or digitalis) glycosides, effects on the myocardium |
| Medication Prescriptions | DILTIAZEM CAP 120MG CD | a calcium channel blocker | angina; hypertension | works by affecting the movement of calcium into the cells of the heart and blood vessels |
| Medication Prescriptions | DILTIAZEM CAP 120MG ER | a calcium channel blocker | angina; hypertension | works by affecting the movement of calcium into the cells of the heart and blood vessels |
| Medication Prescriptions | DILTIAZEM CAP 240MG ER | a calcium channel blocker | angina; hypertension | works by affecting the movement of calcium into the cells of the heart and blood vessels |
| Medication Prescriptions | DILTIAZEM 24HR ER 240 MG CAP | a calcium channel blocker | angina; hypertension | works by affecting the movement of calcium into the cells of the heart and blood vessels |
| Medication Prescriptions | DIOVAN | an angiotensin II receptor blocker (ARB) | hypertension; heart failure and left ventricular failure after a heart attack | works by blocking a substance in the body that causes blood vessels to tighten |
| Medication Prescriptions | DIOVAN 160 MG TABLET | an angiotensin II receptor blocker (ARB) | hypertension; heart failure and left ventricular failure after a heart attack | works by blocking a substance in the body that causes blood vessels to tighten |
| Medication Prescriptions | EPIPEN 0.3 MG AUTO-INJECTOR | a sympathomimetic catecholamine,treatment of emergency allergic reactions | the emergency treatment of allergic reactions (Type I) including anaphylaxis to stinging insects,allergen immunotherapy, foods, drugs, diagnostic testing substances | acts on both alpha and beta adrenergic receptors, lessens the vasodilation and increased vascular permeability that occurs during anaphylaxis, which can lead to loss of intravascular fluid volume and hypotension |
| Medication Prescriptions | EPIPEN 2-PAK INJ 0.3MG | a sympathomimetic catecholamine,treatment of emergency allergic reactions | the emergency treatment of allergic reactions (Type I) including anaphylaxis to stinging insects,allergen immunotherapy, foods, drugs, diagnostic testing substances | acts on both alpha and beta adrenergic receptors, lessens the vasodilation and increased vascular permeability that occurs during anaphylaxis, which can lead to loss of intravascular fluid volume and hypotension |
| Medication Prescriptions | ESCITALOPRAM 10 MG TABLET | an antidepressant, selective serotonin reuptake inhibitors (SSRIs) | depression and generalized anxiety disorder (GAD) | work by increasing the activity of the chemical serotonin in the brain |
| Medication Prescriptions | ESCITALOPRAM 20 MG TABLET | an antidepressant, selective serotonin reuptake inhibitors (SSRIs) | depression and generalized anxiety disorder (GAD) | work by increasing the activity of the chemical serotonin in the brain |
| Medication Prescriptions | ESCITALOPRAM TAB 10MG | an antidepressant, selective serotonin reuptake inhibitors (SSRIs) | depression and generalized anxiety disorder (GAD) | work by increasing the activity of the chemical serotonin in the brain |
| Medication Prescriptions | ETODOLAC TAB 400MG | a nonsteroidal anti-inflammatory drug (NSAID) | mild to moderate pain; arthritis (osteoarthritis and rheumatoid arthritis) | related to prostaglandin synthetase inhibition |
| Medication Prescriptions | FAMOTIDINE TAB 40MG | histamine H2-receptor antagonists or H2-blockers | stomach ulcers (gastric and duodenal), erosive esophagitis (heartburn or acid indigestion), and gastroesophageal reflux disease (GERD) | works by decreasing the amount of acid produced by the stomach |
| Medication Prescriptions | FENOFIBRATE 160 MG TABLET | treat high cholesterol and triglyceride (fat-like substances) levels in the blood; help prevent the development of pancreatitis (inflammation or swelling of the pancreas) caused by high levels of triglycerides in the blood | Primary Hypercholesterolemia or Mixed Dyslipidemia;Severe Hypertriglyceridemia; | raising HDL-C or lowering triglycerides (TG) on the risk of cardiovascular morbidity and mortality |
| Medication Prescriptions | FENTANYL 50 MCG/HR PATCH | relieve moderate to severe chronic pain when around-the-clock pain relief is needed for a long period of time | breakthrough pain in cancer patients | narcotic analgesics (pain medicines), acts on the central nervous system (CNS) to relieve pain |
| Medication Prescriptions | FINASTERIDE TAB 5MG | increase testosterone levels in the body, which decreases prostate size and increases hair growth on the scalp | treat men with benign prostatic hyperplasia (BPH) and male pattern hair loss(androgenetic alopecia) | blocks the action of an enzyme called 5-alpha-reductase. This enzyme changes testosterone to another hormone that causes the prostate to grow or hair loss in males. |
| Medication Prescriptions | FINASTERIDE 5 MG TABLET | increase testosterone levels in the body, which decreases prostate size and increases hair growth on the scalp | treat men with benign prostatic hyperplasia (BPH) and male pattern hair loss(androgenetic alopecia) | blocks the action of an enzyme called 6-alpha-reductase. This enzyme changes testosterone to another hormone that causes the prostate to grow or hair loss in males. |
| Medication Prescriptions | FLUOXETINE CAP 20MG | an antidepressant ; belongs to a group of medicines known as selective serotonin reuptake inhibitors (SSRIs) | depression, obsessive-compulsive disorder (OCD), bulimia nervosa, premenstrual dysphoric disorder (PMDD), and panic disorder | works by increasing the activity of a chemical called serotonin in the brain |
| Medication Prescriptions | FLUTICASONE | Fluticasone propionate is a synthetic trifluorinated corticosteroid | Asthma | Fluticasone propionate is a synthetic trifluorinated corticosteroid with potent anti-inflammatory activity |
| Medication Prescriptions | FLUTICASONE SPR 50MCG | Fluticasone propionate is a synthetic trifluorinated corticosteroid | Asthma | Fluticasone propionate is a synthetic trifluorinated corticosteroid with potent anti-inflammatory activity |
| Medication Prescriptions | FLUZONE HIGH-DOSE 2012-13 SYR | an inactivated influenza virus vaccine; | FLU | active immunization against influenza disease caused by influenza virus subtypes A and type B contained in the vaccine |
| Medication Prescriptions | FOLIC ACID 1 MG TABLET | a B vitamin (B9) | early pregnancy |  |
| Medication Prescriptions | FUROSEMIDE TAB 20MG | help treat fluid retention and swelling that is caused by congestive heart failure, liver disease, kidney disease, or other medical conditions | edema,congestive heart failure, liver disease, kidney disease,hypertension | works by acting on the kidneys to increase the flow of urine |
| Medication Prescriptions | FUROSEMIDE TAB 40MG | help treat fluid retention and swelling that is caused by congestive heart failure, liver disease, kidney disease, or other medical conditions | edema,congestive heart failure, liver disease, kidney disease,hypertension | works by acting on the kidneys to increase the flow of urine |
| Medication Prescriptions | FUROSEMIDE 80 MG TABLET | help treat fluid retention and swelling that is caused by congestive heart failure, liver disease, kidney disease, or other medical conditions | edema,congestive heart failure, liver disease, kidney disease,hypertension | works by acting on the kidneys to increase the flow of urine |
| Medication Prescriptions | GABAPENTIN CAP 300MG | control seizures ;not cure epilepsy; an anticonvulsant | epilepsy;postherpetic neuralgia;Restless Legs Syndrome (RLS) | works in the brain to prevent seizures and relieve pain for certain conditions in the nervous system |
| Medication Prescriptions | GABAPENTIN CAP 400MG | control seizures ;not cure epilepsy; an anticonvulsant | epilepsy;postherpetic neuralgia;Restless Legs Syndrome (RLS) | works in the brain to prevent seizures and relieve pain for certain conditions in the nervous system |
| Medication Prescriptions | HYDROCHLOROT | a thiazide diuretic;reduce the amount of water in the body by increasing the flow of urine | hypertension;edema; congestive heart failure, severe liver disease (cirrhosis), kidney disease | affect the renal tubular mechanisms of electrolyte reabsorption, directly increasing excretion of sodium and chloride in approximately equivalent amounts |
| Medication Prescriptions | HYDROCODON-ACETAMINOPH 7.5-325 | an opioid analgesic and antitussive | the relief of moderate to moderately severe pain | the central nervous system and smooth muscle |
| Medication Prescriptions | HYDROCODON-ACETAMINOPHEN 5-500 | an opioid analgesic and antitussive | the relief of moderate to moderately severe pain | the central nervous system and smooth muscle |
| Medication Prescriptions | HYDROCODONE-ACETAMINOPHEN 5MG-3 | an opioid analgesic and antitussive | the relief of moderate to moderately severe pain | the central nervous system and smooth muscle |
| Medication Prescriptions | HYDROCODONE-APAP 5-500 TABLET | an opioid analgesic and antitussive | the relief of moderate to moderately severe pain | the central nervous system and smooth muscle |
| Medication Prescriptions | HYDROXYZ PAM CAP 50MG | relief of anxiety and tension | psychoneurosis ; pruritus | activity in certain key regions of the subcortical area of the central nervous system |
| Medication Prescriptions | HYDROXYZINE PAM 25 MG CAP | not a cortical depressant | anxiety , tension, pruritus | a suppression of activity in certain key regions of the subcortical area of the central nervous system |
| Medication Prescriptions | IBUPROFEN TAB 800MG | nonsteroidal anti-inflammatory drug (NSAID) | mild to moderate pain, arthritis,fever, menstrual cramps | related to prostaglandin synthetase inhibition |
| Medication Prescriptions | IBUPROFEN 600 MG TABLET | nonsteroidal anti-inflammatory drug (NSAID) | mild to moderate pain, arthritis,fever, menstrual cramps | related to prostaglandin synthetase inhibition |
| Medication Prescriptions | IPRAT-ALBUT 0.5-3(2.5) MG/3 ML | treat air flow blockage and prevent the worsening of chronic obstructive pulmonary disease | asthma, chronic bronchitis, and emphysema | a combination of the β2-adrenergic bronchodilator,open up the bronchial tubes (air passages) in the lungs |
| Medication Prescriptions | IPRATROPIUM/ SOL ALBUTER | treat air flow blockage and prevent the worsening of chronic obstructive pulmonary disease | asthma, chronic bronchitis, and emphysema | a combination of the β3-adrenergic bronchodilator,open up the bronchial tubes (air passages) in the lungs |
| Medication Prescriptions | ISOSORB MONO TAB 120MG ER | prevention of angina pectoris due to coronary artery disease | coronary artery disease | nitrates, works by relaxing the blood vessels and increasing the supply of blood and oxygen to the heart while reducing its work load |
| Medication Prescriptions | JANTOVEN TAB 1MG | an anticoagulant | venous thrombosis and its extension, and pulmonary embolism | inhibiting vitamin K-dependent coagulation factors |
| Medication Prescriptions | KETOROLAC 10 MG TABLET | nonsteroidal anti-inflammatory drugs (NSAIDs) | Acute Pain | related to prostaglandin synthetase inhibition |
| Medication Prescriptions | KLOR-CON(potassium chloride) M10 TABLET | a solid oral dosage form of potassium chloride | hypokalemia |  |
| Medication Prescriptions | LABETALOL TAB 200MG | fall in diastolic blood pressure | hypertension | adrenergic receptor blocking agents that have both selective alpha1-adrenergic and nonselective beta-adrenergic receptor blocking actions in a single substance |
| Medication Prescriptions | LAMOTRIGINE TAB 25MG | an AED of the phenyltriazine class | seizures;epilepsy;bipolar disorder | Unknown, preventing seizure spread in the maximum electroshock (MES) and pentylenetetrazol (scMet) tests, and prevented seizures in the visually and electrically evoked after-discharge (BEAD) tests for antiepileptic activity |
| Medication Prescriptions | LAMOTRIGINE 25 MG TABLET | an AED of the phenyltriazine class | seizures;epilepsy;bipolar disorder | Unknown, preventing seizure spread in the maximum electroshock (MES) and pentylenetetrazol (scMet) tests, and prevented seizures in the visually and electrically evoked after-discharge (BEAD) tests for antiepileptic activity |
| Medication Prescriptions | LANSOPRAZOLE CAP 30MG DR | treat certain conditions in which there is too much acid in the stomach | erosive esophagitis ; "heartburn" caused by gastroesophageal reflux disease (GERD) | is a proton pump inhibitor (PPI). works by decreasing the amount of acid produced by the stomach |
| Medication Prescriptions | LANSOPRAZOLE DR 30 MG CAPSULE | treat certain conditions in which there is too much acid in the stomach | erosive esophagitis ; "heartburn" caused by gastroesophageal reflux disease (GERD) | is a proton pump inhibitor (PPI). works by decreasing the amount of acid produced by the stomach |
| Medication Prescriptions | LEVOFLOXACIN 500 MG TABLET | quinolone antibiotics | bacterial infections | killing bacteria or preventing their growth |
| Medication Prescriptions | LEVOFLOXACIN TAB 500MG | quinolone antibiotics | bacterial infections | killing bacteria or preventing their growth |
| Medication Prescriptions | LEVOTHYROXIN TAB 175MCG | prevention of various types of euthyroid goiters | hypothyroidism;thyroid cancer | regulated by the hypothalamic-pituitary-thyroid axis; through control of DNA transcription and protein synthesis |
| Medication Prescriptions | LEVOTHYROXIN TAB 25MCG | prevention of various types of euthyroid goiters | hypothyroidism;thyroid cancer | regulated by the hypothalamic-pituitary-thyroid axis; through control of DNA transcription and protein synthesis |
| Medication Prescriptions | LEVOTHYROXINE 100 MCG TABLET | prevention of various types of euthyroid goiters | hypothyroidism;thyroid cancer | regulated by the hypothalamic-pituitary-thyroid axis; through control of DNA transcription and protein synthesis |
| Medication Prescriptions | LEVOTHYROXINE 125 MCG TABLET | prevention of various types of euthyroid goiters | hypothyroidism;thyroid cancer | regulated by the hypothalamic-pituitary-thyroid axis; through control of DNA transcription and protein synthesis |
| Medication Prescriptions | LEVOTHYROXINE 175 MCG TABLET | prevention of various types of euthyroid goiters | hypothyroidism;thyroid cancer | regulated by the hypothalamic-pituitary-thyroid axis; through control of DNA transcription and protein synthesis |
| Medication Prescriptions | LEVOTHYROXINE 200 MCG TABLET | prevention of various types of euthyroid goiters | hypothyroidism;thyroid cancer | regulated by the hypothalamic-pituitary-thyroid axis; through control of DNA transcription and protein synthesis |
| Medication Prescriptions | LEVOTHYROXINE 25 MCG TABLET | prevention of various types of euthyroid goiters | hypothyroidism;thyroid cancer | regulated by the hypothalamic-pituitary-thyroid axis; through control of DNA transcription and protein synthesis |
| Medication Prescriptions | LIDODERM DIS 5% | an amide-type local anesthetic agent | relief of pain associated with post-herpetic neuralgia | stabilize neuronal membranes by inhibiting the ionic fluxes required for the initiation and conduction of impulses |
| Medication Prescriptions | LIPITOR (Atorvastatin)80 MG TABLET | lowers the level of cholesterol in the blood | prevent certain types of heart and blood vessel problems in patients with risk factors for heart problems | inhibitor of 3-hydroxy-3-methylglutaryl-coenzyme A (HMG-CoA) reductase . This enzyme catalyzes the conversion of HMG-CoA to mevalonate, an early and rate-limiting step in cholesterol biosynthesis. |
| Medication Prescriptions | LISINOP/HCTZ (lisinopril and hydrochlorothiazide)TAB 10-12.5 | treat high blood pressure | hypertesion | Lisinopril is angiotensin-converting enzyme (ACE) inhibitors. It works by decreasing certain chemicals that tighten the blood vessels, so blood flows more smoothly. Hydrochlorothiazide is in a diuretics. It works by causing the kidneys to get rid of unneeded water and salt from the body into the urine. |
| Medication Prescriptions | LISINOP/HCTZ TAB 20-12.5 | treat high blood pressure | hypertesion | Lisinopril is angiotensin-converting enzyme (ACE) inhibitors. It works by decreasing certain chemicals that tighten the blood vessels, so blood flows more smoothly. Hydrochlorothiazide is in a diuretics. It works by causing the kidneys to get rid of unneeded water and salt from the body into the urine. |
| Medication Prescriptions | LISINOPRIL TAB 10MG | treat high blood pressure; lowers blood pressure and increases the supply of blood and oxygen to the heart | hypertesion;heart failure;heart attack | works by blocking a substance in the body that causes the blood vessels to tighten |
| Medication Prescriptions | LISINOPRIL TAB 20MG | treat high blood pressure; lowers blood pressure and increases the supply of blood and oxygen to the heart | hypertesion;heart failure;heart attack | works by blocking a substance in the body that causes the blood vessels to tighten |
| Medication Prescriptions | LISINOPRIL 40 MG TABLET | treat high blood pressure; lowers blood pressure and increases the supply of blood and oxygen to the heart | hypertesion;heart failure;heart attack | works by blocking a substance in the body that causes the blood vessels to tighten |
| Medication Prescriptions | LORAZEPAM TAB 0.5MG | benzodiazepine | anxiety | central nervous system (CNS) depressants, which are medicines that slow down the nervous system |
| Medication Prescriptions | LORAZEPAM 0.5MG TABLET | benzodiazepine | anxiety | central nervous system (CNS) depressants, which are medicines that slow down the nervous system |
| Medication Prescriptions | LOSARTAN POT TAB 25MG | treat high blood pressure | hypertesion | an angiotensin II receptor (type AT1) antagonist |
| Medication Prescriptions | LOSARTAN POT TAB 50MG | treat high blood pressure | hypertesion | an angiotensin II receptor (type AT2) antagonist |
| Medication Prescriptions | LOSARTAN POTASSIUM 50 MG TAB | treat high blood pressure | hypertesion | an angiotensin II receptor (type AT3) antagonist |
| Medication Prescriptions | LOVASTATIN TAB 20MG | lower cholesterol and triglyceride (fat) levels in the blood | chest pain, heart attack, stroke | HMG-CoA reductase inhibitors or statins. It works to reduce the amount of cholesterol in the blood by blocking an enzyme that is needed by the body to make cholesterol |
| Medication Prescriptions | LOVASTATIN 40 MG TABLET | lower cholesterol and triglyceride (fat) levels in the blood | chest pain, heart attack, stroke | HMG-CoA reductase inhibitors or statins. It works to reduce the amount of cholesterol in the blood by blocking an enzyme that is needed by the body to make cholesterol |
| Medication Prescriptions | METHYLPRED PAK 4MG | glucocorticoid | Endocrine Disorders; Rheumatic Disorders; Collagen Diseases; Dermatologic Diseases; Allergic States; Ophthalmic Diseases; Respiratory Diseases; Hematologic Disorders; Neoplastic Diseases; Edematous States; Gastrointestinal Diseases;Nervous System; Miscellaneous | potent anti-inflammatory effects |
| Medication Prescriptions | METHYLPREDNISOLONE 4 MG DOSEPK | a glucocorticoid | Endocrine Disorders;Rheumatic Disorders;Collagen Diseases;Dermatologic Diseases;Allergic States;Ophthalmic Diseases;Respiratory Diseases; Hematologic Disorders; Neoplastic Diseases; Edematous States; Gastrointestinal Diseases; Nervous System; Miscellaneous | as replacement therapy in adrenocortical deficiency states; anti-inflammatory effects |
| Medication Prescriptions | METOCLOPRAMIDE 10 MG TABLET | increases the resting tone of the lower esophageal sphincter | Symptomatic Gastroesophageal Reflux;Diabetic Gastroparesis (Diabetic Gastric Stasis) | stimulates motility of the upper gastrointestinal tract without stimulating gastric, biliary, or pancreatic secretions |
| Medication Prescriptions | METOLAZONE 2.5 MG TABLET | a quinazoline diuretic | hypertension | inhibit sodium reabsorption at the cortical diluting site and to a lesser extent in the proximal convoluted tubule |
| Medication Prescriptions | METOPROL TAR | the heart beats slower and decreases the blood pressure | acute heart attack | a beta-blocker. It works by affecting the response to nerve impulses in certain parts of the body, like the heart |
| Medication Prescriptions | METOPROLOL TAB 25MG ER | effect on beta1 adrenoreceptors, chiefly located in cardiac muscle; inhibits beta2 adrenoreceptors, chiefly located in the bronchial and vascular musculature | Hypertension;Angina Pectoris;Myocardial Infarction | a selective betai-adrenoreceptor blocking agent |
| Medication Prescriptions | METOPROLOL TAB 50MG ER | effect on beta1 adrenoreceptors, chiefly located in cardiac muscle; inhibits beta3 adrenoreceptors, chiefly located in the bronchial and vascular musculature | Hypertension;Angina Pectoris;Myocardial Infarction | a selective betai-adrenoreceptor blocking agent |
| Medication Prescriptions | METOPROLOL SUCC ER 25 MG TAB | effect on beta1 adrenoreceptors, chiefly located in cardiac muscle; inhibits beta4 adrenoreceptors, chiefly located in the bronchial and vascular musculature | Hypertension;Angina Pectoris;Myocardial Infarction | a selective betai-adrenoreceptor blocking agent |
| Medication Prescriptions | METOPROLOL SUCC ER 50MG TAB | effect on beta1 adrenoreceptors, chiefly located in cardiac muscle; inhibits beta5 adrenoreceptors, chiefly located in the bronchial and vascular musculature | Hypertension;Angina Pectoris;Myocardial Infarction | a selective betai-adrenoreceptor blocking agent |
| Medication Prescriptions | METOPROLOL TARTRATE 100 MG TAB | effect on beta1 adrenoreceptors, chiefly located in cardiac muscle; inhibits beta6 adrenoreceptors, chiefly located in the bronchial and vascular musculature | Hypertension;Angina Pectoris;Myocardial Infarction | a selective betai-adrenoreceptor blocking agent |
| Medication Prescriptions | METOPROLOL TARTRATE 50MG TAB | effect on beta1 adrenoreceptors, chiefly located in cardiac muscle; inhibits beta7 adrenoreceptors, chiefly located in the bronchial and vascular musculature | Hypertension;Angina Pectoris;Myocardial Infarction | a selective betai-adrenoreceptor blocking agent |
| Medication Prescriptions | MIRTAZAPINE TAB 45MG | tetracyclic antidepressants | depression | works in the central nervous system (CNS) to make certain chemicals in the brain stronger |
| Medication Prescriptions | MONTELUKAST SOD 10 MG TABLET | Montelukast inhibits physiologic actions of LTD4 at the CysLT1 receptor without any agonist activity | Asthma;Exercise-Induced Bronchoconstriction (EIB);Allergic Rhinitis | Montelukast inhibits physiologic actions of LTD4 at the CysLT1 receptor without any agonist activity |
| Medication Prescriptions | MUPIROCIN 2% OINTMENT | Each gram of Bactroban Ointment (mupirocin ointment) | impetigo | against a wide range of gram-positive bacteria including methicillin-resistant Staphylococcus aureus (MRSA) |
| Medication Prescriptions | NABUMETONE 500 MG TABLET | naphthylalkanone | relief of signs and symptoms of osteoarthritis and rheumatoid arthritis | a non-steroidal anti-inflammatory drug (NSAID) that exhibits anti-inflammatory, analgesic, and antipyretic properties |
| Medication Prescriptions | NICOTINE 21 MG/24HR PATCH | Nicotine is one of the most heavily used addictive drugs in the U.S., and the leading preventable cause of disease, disability, and death in the U.S. | quit smoking; psychological treatment | acts on the brain; nicotine increases the levels of dopamine |
| Medication Prescriptions | NITROFURANTN CAP 100MG | an antibiotic | urinary tract infections | killing bacteria or preventing their growth |
| Medication Prescriptions | NITROFURANTOIN MONO-MCR 100 MG | an antibacterial | urinary tract infections | killing bacteria or preventing their growth |
| Medication Prescriptions | NOVOLOG INJ 100/ML | a rapid-acting human insulin |  |  |
| Medication Prescriptions | NOVOLOG INJ FLEXPEN | a rapid-acting human insulin |  |  |
| Medication Prescriptions | NOVOLOG INJ FLEXPEN | a rapid-acting human insulin |  |  |
| Medication Prescriptions | NOVOLOG 100 UNIT/ML VIAL | a rapid-acting human insulin |  |  |
| Medication Prescriptions | NOVOLOG FLEXPEN SYRINGE | a rapid-acting human insulin |  |  |
| Medication Prescriptions | NYSTATIN 100,000 UNIT/GM CREAM | an antimycotic poly-ene antibiotic | candidiasis | binding to sterols in the cell membrane of susceptible Candida species with a resultant change in membrane permeability allowing leakage of intracellular components |
| Medication Prescriptions | NYSTATIN 100,000 UNITS/ML SUSP | an antimycotic poly-ene antibiotic | candidiasis | binding to sterols in the cell membrane of susceptible Candida species with a resultant change in membrane permeability allowing leakage of intracellular components |
| Medication Prescriptions | NYSTOP POW 100000 | a polyene antifungal antibiotic | cutaneous or mucocutaneous mycotic infections caused by Candida albicans and other susceptible Candida species | against a wide variety of yeasts and yeast-like fungi, binding to sterols in the cell membrane of susceptible species resulting in a change in membrane permeability and the subsequent leakage of intracellular components. |
| Medication Prescriptions | NYSTOP 100,000 UNITS/GM POWDER | a polyene antifungal antibiotic | cutaneous or mucocutaneous mycotic infections caused by Candida albicans and other susceptible Candida species | against a wide variety of yeasts and yeast-like fungi, binding to sterols in the cell membrane of susceptible species resulting in a change in membrane permeability and the subsequent leakage of intracellular components. |
| Medication Prescriptions | OLANZAPINE 2.5 MG TABLET | an atypical antipsychotic | schizophrenia | an antagonist with moderate affinity binding for serotonin 5HT3 and muscarinic M1-5 |
| Medication Prescriptions | OLANZAPINE 5 MG TABLET | an atypical antipsychotic | schizophrenia | an antagonist with moderate affinity binding for serotonin 5HT3 and muscarinic M1-6 |
| Medication Prescriptions | OXCARBAZEPIN TAB 300MG | an antiepileptic drug | seizures; epilepsy | blockade of voltage-sensitive sodium channels,resulting in stabilization of hyperexcited neural membranes, inhibition of repetitive neuronal firing, and diminution of propagation of synaptic impulses. |
| Medication Prescriptions | OXYBUTYNIN TAB 10MG ER | decrease muscle spasms of the bladder and the frequent urge to urinate caused by these spasms | relief of symptoms of bladder instability | direct antispasmodic effect on smooth muscle and inhibits the muscarinic action of acetylcholine on smooth muscle. |
| Medication Prescriptions | OXYBUTYNIN TAB 5MG | decrease muscle spasms of the bladder and the frequent urge to urinate caused by these spasms | relief of symptoms of bladder instability | direct antispasmodic effect on smooth muscle and inhibits the muscarinic action of acetylcholine on smooth muscle. |
| Medication Prescriptions | OXYBUTYNIN 5 MG TABLET | decrease muscle spasms of the bladder and the frequent urge to urinate caused by these spasms | relief of symptoms of bladder instability | direct antispasmodic effect on smooth muscle and inhibits the muscarinic action of acetylcholine on smooth muscle. |
| Medication Prescriptions | OXYCODAPAP | semisynthetic opioid analgesic | relief of moderate to moderately severe pain. | the inhibition of endogenous pyrogen action on the hypothalamic heat-regulating centers. |
| Medication Prescriptions | OXYCODONE TAB 15MG | semisynthetic opioid analgesic | relief of moderate to moderately severe pain. | the inhibition of endogenous pyrogen action on the hypothalamic heat-regulating centers. |
| Medication Prescriptions | OXYCODONE HCL 30 MG TABLET | semisynthetic opioid analgesic | relief of moderate to moderately severe pain. | the inhibition of endogenous pyrogen action on the hypothalamic heat-regulating centers. |
| Medication Prescriptions | OXYCODONE-ACETAMINOPHEN 5-325 | semisynthetic opioid analgesic | relief of moderate to moderately severe pain. | the inhibition of endogenous pyrogen action on the hypothalamic heat-regulating centers. |
| Medication Prescriptions | PAROXETINE | administered psychotropic drug | major depressive disorder | paroxetine blocks the uptake of serotonin into human platelets. |
| Medication Prescriptions | PENICILLIN VK 500 MG TABLET | the phenoxymethyl analog of penicillin G | different types of infections caused by bacteria | exerts a bactericidal action against penicillin-sensitive microorganisms during the stage of active multiplication |
| Medication Prescriptions | POLYETH GLYC POW 3350 NF(polyethylene glycol ) | is as osmotic agent which causes water to be retained with the stool | occasional constipation | is as osmotic agent which causes water to be retained with the stool |
| Medication Prescriptions | POT CL MICRO ( Potassium Chloride (KCl) | microencapsulation and a dispersing agent | hypokalemia | supplementation |
| Medication Prescriptions | PRAVASTATIN SODIUM 40 MG TAB | reduce cholesterol biosynthesis | Prevention of Cardiovascular Disease; Hyperlipidemia | a reversible inhibitor of 3-hydroxy-3-methylglutaryl-coenzyme A (HMG-CoA) reductase, the enzyme that catalyzes the conversion of HMG-CoA to mevalonate, an early and rate limiting step in the biosynthetic pathway for cholesterol |
| Medication Prescriptions | PREDNISONE TAB 20MG | contain prednisone which is a glucocorticoid | Endocrine Disorders; Rheumatic Disorders; Collagen Diseases; Dermatologic Diseases; Allergic States; Ophthalmic Diseases; Respiratory Diseases; Hematologic Disorders; Neoplastic Diseases; Edematous States; Gastrointestinal Diseases; Nervous System; Miscellaneous | are used as replacement therapy in adrenocortical deficiency states; used for their potent anti-inflammatory effects in disorders of many organ systems |
| Medication Prescriptions | PREDNISONE TAB 5MG | contain prednisone which is a glucocorticoid | Endocrine Disorders; Rheumatic Disorders; Collagen Diseases; Dermatologic Diseases; Allergic States; Ophthalmic Diseases; Respiratory Diseases; Hematologic Disorders; Neoplastic Diseases; Edematous States; Gastrointestinal Diseases; Nervous System; Miscellaneous | are used as replacement therapy in adrenocortical deficiency states; used for their potent anti-inflammatory effects in disorders of many organ systems |
| Medication Prescriptions | PREDNISONE 20 MG TABLET | contain prednisone which is a glucocorticoid | Endocrine Disorders; Rheumatic Disorders; Collagen Diseases; Dermatologic Diseases; Allergic States; Ophthalmic Diseases; Respiratory Diseases; Hematologic Disorders; Neoplastic Diseases; Edematous States; Gastrointestinal Diseases; Nervous System; Miscellaneous | are used as replacement therapy in adrenocortical deficiency states; used for their potent anti-inflammatory effects in disorders of many organ systems |
| Medication Prescriptions | PROAIR HFA(albuterol sulfate) | a beta2-adrenergic agonist | Bronchospasm; Exercise-Induced Bronchospasm | activation of beta2-adrenergic receptors on airway smooth muscle |
| Medication Prescriptions | PROAIR HFA(albuterol sulfate) 90MCG INHALER | a beta3-adrenergic agonist | Bronchospasm; Exercise-Induced Bronchospasm | activation of beta3-adrenergic receptors on airway smooth muscle |
| Medication Prescriptions | PROCHLORPERAZINE 10 MG TAB | a phenothiazine derivative | nausea and vomiting; schizophrenia; generalized non-psychotic anxiety | exerts an antiemetic effect through a depressant action on the chemoreceptor trigger zone |
| Medication Prescriptions | QUETIAPINE (quetiapine fumarate) TAB 25MG | a psychotropic agent | Schizophrenia;Bipolar Disorder; Special Considerations in Treating Pediatric Schizophrenia and Bipolar I Disorder | unknow, through a combination of dopamine type 2 (D2) and serotonin type 2 (5HT2) antagonism |
| Medication Prescriptions | RESTASIS 0.05% EYE EMULSION | It is an immunosuppressive agent | treat chronic dry eye that may be caused by inflammation | suppress ocular inflammation |
| Medication Prescriptions | ROPINIROLE TAB 0.5MG | an orally administered non-ergoline dopamine agonist | Parkinson's Disease; Restless Legs Syndrome | specificity and full intrinsic activity at the D2 and D3 dopamine receptor subtypes, binding with higher affinity to D3 than to D2 or D4 receptor subtypes |
| Medication Prescriptions | ROPINIROLE TAB 1MG | an orally administered non-ergoline dopamine agonist | Parkinson's Disease; Restless Legs Syndrome | specificity and full intrinsic activity at the D2 and D3 dopamine receptor subtypes, binding with higher affinity to D3 than to D2 or D5 receptor subtypes |
| Medication Prescriptions | SEA-OMEGA(omega-3 polyunsaturated fatty acids) 50 CAPSULE | certain types of fish, vegetables, and other plant sources; not made by the body and must be consumed in the diet | High levels of triglycerides | lower triglyceride levels in the blood |
| Medication Prescriptions | SERTRALINE TAB 100MG | selective serotonin reuptake inhibitors (SSRIs) | depression, obsessive-compulsive disorder (OCD), panic disorder, premenstrual dysphoric disorder (PMDD), posttraumatic stress disorder (PTSD), and social anxiety disorder (SAD) | works by increasing the activity of a chemical called serotonin in the brain |
| Medication Prescriptions | SINGULAIR(MONTELUKAST SOD) 10 MG TABLET | Montelukast inhibits physiologic actions of LTD4 at the CysLT1 receptor without any agonist activity | Asthma;Exercise-Induced Bronchoconstriction (EIB);Allergic Rhinitis | Montelukast inhibits physiologic actions of LTD4 at the CysLT1 receptor without any agonist activity |
| Medication Prescriptions | SOTALOL 80 MG TABLET | an antiarrhythmic drug with Class II (beta-adrenoreceptor blocking) and Class III (cardiac action potential duration prolongation) properties | ventricular arrhythmias;hypertension |  |
| Medication Prescriptions | SPIRIVA(tiotropium bromide) | bronchodilators,open up the bronchial tubes (air passages) in the lungs | bronchospasm or wheezing caused by chronic obstructive pulmonary disease (COPD) | increase the flow of air to the lungs |
| Medication Prescriptions | SULFAMETHOXAZOLE-TMP DS TABLET(sulfamethoxazole and trimethoprim) | different types of infection caused by bacteria | ear infections, urinary tract infections, bronchitis, traveler's diarrhea,shigellosis, and Pneumocystis jiroveci pneumonia | a synthetic antibacterial combination;Sulfamethoxazole inhibits bacterial synthesis of dihydrofolic acid by competing with paraaminobenzoic acid (PABA). Trimethoprim blocks the production of tetrahydrofolic acid from dihydrofolic acid by binding to and reversibly inhibiting the required enzyme, dihydrofolate reductase |
| Medication Prescriptions | SULFAMETHOXAZOLE-TMP SS TABLET(sulfamethoxazole and trimethoprim) | different types of infection caused by bacteria | ear infections, urinary tract infections, bronchitis, traveler's diarrhea,shigellosis, and Pneumocystis jiroveci pneumonia | a synthetic antibacterial combination;Sulfamethoxazole inhibits bacterial synthesis of dihydrofolic acid by competing with paraaminobenzoic acid (PABA). Trimethoprim blocks the production of tetrahydrofolic acid from dihydrofolic acid by binding to and reversibly inhibiting the required enzyme, dihydrofolate reductase |
| Medication Prescriptions | SUMATRIPTAN TAB 100MG | selective serotonin receptor agonists | migraine headaches | Sumatriptan binds with high affinity to human cloned 5-HT1B/1D receptors; agonist effects at the 5-HT1B/1D receptors on intracranial blood vessels and sensory nerves of the trigeminal system, which result in cranial vessel constriction and inhibition of pro-inflammatory neuropeptide release |
| Medication Prescriptions | SYMBICORT AER 160-4.5(budesonide and formoterol fumarate dihydrate) | a long period of time to treat asthma | Maintenance Treatment of Asthma | Budesonide is an anti-inflammatory corticosteroid that exhibits potent glucocorticoid activity and weak mineralocorticoid activity.Formoterol fumarate is a long-acting selective beta2-adrenergic agonist (beta2-agonist) with a rapid onset of action |
| Medication Prescriptions | SYMBICORT 160-4.5 MCG INHALER(budesonide and formoterol fumarate dihydrate) | a long period of time to treat asthma | Maintenance Treatment of Asthma | Budesonide is an anti-inflammatory corticosteroid that exhibits potent glucocorticoid activity and weak mineralocorticoid activity.Formoterol fumarate is a long-acting selective beta2-adrenergic agonist (beta3-agonist) with a rapid onset of action |
| Medication Prescriptions | TAMSULOSIN (Tamsulosin hydrochloride) CAP 0.4MG | an antagonist of alpha1A adrenoceptors in the prostate | the signs and symptoms of benign prostatic hyperplasia (BPH) ;hypertension | Blockade of these adrenoceptors can cause smooth muscles in the bladder neck and prostate to relax, resulting in an improvement in urine flow rate and a reduction in symptoms of BPH. |
| Medication Prescriptions | TRAMADOL HCL TAB 50MG | a centrally acting analgesic | moderate to moderately severe pain in adults | binding of parent and M1 metabolite to μ-opioid receptors and weak inhibition of reuptake of norepinephrine and serotonin |
| Medication Prescriptions | TRAZODONE | an antidepressant | depression; dysphoric mood | selectively inhibits serotonin uptake by brain synaptosomes and potentiates the behavioral changes induced by the serotonin precursor, 5-hydroxytryptophan |
| Medication Prescriptions | TRAZODONE 100 MG TABLET | an antidepressant | depression; dysphoric mood | selectively inhibits serotonin uptake by brain synaptosomes and potentiates the behavioral changes induced by the serotonin precursor, 6-hydroxytryptophan |
| Medication Prescriptions | TRAZODONE 150 MG TABLET | an antidepressant | depression; dysphoric mood | selectively inhibits serotonin uptake by brain synaptosomes and potentiates the behavioral changes induced by the serotonin precursor, 7-hydroxytryptophan |
| Medication Prescriptions | TRIAMCINOLONE 0.1% PASTE | a class of primarily synthetic steroids used as anti-inflammatory and antipruritic agents | relief of the inflammatory and pruritic manifestations of corticosteroid-responsive dermatoses | The mechanism of anti-inflammatory activity of the topical corticosteroids is unclear. |
| Medication Prescriptions | TRICOR (Fenofibrate) TAB 145MG | treat high cholesterol and triglyceride (fat-like substances) levels in the blood; help prevent the development of pancreatitis (inflammation or swelling of the pancreas) caused by high levels of triglycerides in the blood | Primary Hypercholesterolemia or Mixed Dyslipidemia;Severe Hypertriglyceridemia; | raising HDL-C or lowering triglycerides (TG) on the risk of cardiovascular morbidity and mortality |
| Medication Prescriptions | VENLAFAXINE CAP 150MG ER | novel antidepressant | depressive disorder | its potentiation of neurotransmitter activity in the CNS |
| Medication Prescriptions | VENLAFAXINE HCL ER 75 MG CAP | novel antidepressant | depressive disorder | its potentiation of neurotransmitter activity in the CNS |
| Medication Prescriptions | VENTOLIN HFA AER | prevention of bronchospasm | Bronchospasm | preferential effect on beta2-adrenergic receptors |
| Primary Diagnosis | LONGTERM USE ANTICOAG (Begin 1995) |  |  |  |
| Radiology Test | Chest Narrative X-ray 2 views-Normal |  |  |  |
| Radiology Test | Chest Narrative X-ray portable-Normal |  |  |  |
| Radiology Test | Head Narrative CT WO contrast-Normal |  |  |  |
