# Supplementary material for: Defining and characterizing the critical transition state prior to the type 2 diabetes disease
Source: PLoS One. 2017 Jul 7;12(7):e0180937. doi: 10.1371/journal.pone.0180937 (PMC5501620; doi:10.1371/journal.pone.0180937)
Supplement: S2 Table — (DOCX) [file pone.0180937.s004.docx]

**S2 Table. The demographic characteristics of the case and control cohort.**

|  | Case (N=7334) | Control (N=10,145) |
| --- | --- | --- |
| Average age, years | 62.4 | 62.3 |
| Female, % | 48.4 | 48.4 |
| Asthma, % | 3.1 | 0 |
| Hypertension, % | 17.8 | 0.4 |
| Obesity, % | 0.3 | 0 |
| Disorders of lipid metabolism, % | 15.6 | 0.3 |
| Chronic obstructive pulmonary disease, % | 4.3 | 0.1 |
| Chronic kidney disease, % | 2.2 | 0 |
| Thyroid disorders | 5.4 | 0.1 |
| Cardiac arrhythmias, % | 5.9 | 0.1 |
| Total number of chronic diseases, mean | 1.7 | 0.03 |
| Total costs, USD, mean | 3907.5 | 1350.6 |
